# Selective exosome exclusion of miR-375 by glioma cells promotes glioma progression by activating the *CTGF*-EGFR pathway

**DOI:** 10.1186/s13046-020-01810-9

**Published:** 2021-01-06

**Authors:** Xiangdong Xu, Yang Liu, Yan Li, Huajian Chen, Yuxuan Zhang, Jie Liu, Shaokang Deng, Yaofeng Zheng, Xinlin Sun, Jihui Wang, Taoliang Chen, Min Huang, Yiquan Ke

**Affiliations:** The National Key Clinical Specialty, The Engineering Technology Research Center of Education Ministry of China, Guangdong Provincial Key Laboratory on Brain Function Repair and Regeneration, Department of Neurosurgery, Zhujiang Hospital, Southern Medical University, Guangzhou, 510282 China

**Keywords:** miR-375, Exosome, *Connectivetissuegrowthfactor*, Glioma, Proliferation, Migration, Invasion

## Abstract

**Background:**

Exosomes are membrane-bound extracellular vesicles of 40–150 nm in size, that are produced by many cell types, and play an important role in the maintenance of cellular homeostasis. Exosome secretion allows for the selective removal of harmful substances from cells. However, it remains unclear whether this process also takes place in glioma cells.

**Methods:**

Herein, the role of the tumour-suppressor miR-375 was explored in human glioma cells. Immunoblotting and qRT-PCR experiments demonstrated a functional link between miR-375 and its target, *connective* *tissue* *growth* *factor* (*CTGF*), which led to the identification of the underlying molecular pathways. The exosomes secreted by glioma cells were extracted by ultracentrifugation and examined by transmission electron microscopy. Exosomal expression of miR-375 was then analysed by qRT-PCR; while the exosome secretion inhibitor, GW4869, was used to examine the biological significance of miR-375 release. Moreover, the dynamics of miR-375 release by glioma cells was investigated using fluorescently labelled exosomes. Finally, exosomal miR-375 release was examined in an orthotopic xenograft model in nude mice.

**Results:**

MiR-375 expression was downregulated in gliomas. MiR-375 suppressed glioma proliferation, migration, and invasion by inhibiting the *CTGF*-*epidermal* *growth* *factor* *receptor* (EGFR) signalling pathway. MiR-375-containing exosomes were also identified in human peripheral blood samples from glioma patients, and their level correlated with disease progression status. Exosomal miR-375 secretion impacted the *CTGF*-EGFR pathway activity. Once secreted, exosomal miR-375 was not taken back up by glioma cells.

**Conclusions:**

Exosomal miR-375 secretion allowed for sustained activation of the *CTGF*-EGFR oncogenic pathway, promoting the proliferation and invasion of glioma cells. These findings enhance our understanding of exosome biology and may inspire development of new glioma therapies.

**Supplementary Information:**

The online version contains supplementary material available at 10.1186/s13046-020-01810-9.

## Background

Glioma is an intracranial malignancy and the most prevalent neoplasm of the central nervous system, with an annual incidence of ~ 5/100,000 cases worldwide [1]. According to the World Health Organisation (WHO) classification, gliomas are divided into well-differentiated low-grade astrocytomas (WHO I–II), anaplastic astrocytomas (WHO III), and glioblastoma multiforme (GBM, WHO IV) [2]. GBM represents the most common and lethal type of glioma, with a median survival time from diagnosis of less than 15 months in optimally treated patients, and an overall 5-year survival rate < 5% [3]. Even with the current treatment strategies, including surgery, chemotherapy, radiation therapy, targeted therapy, and immunotherapy, the prognosis of glioma remains unfavourable, with a high recurrence rate after initial treatment [4]. Therefore, characterising the underlying molecular mechanisms and developing novel therapeutic options, are high-priority goals.

MicroRNAs (miRNAs) are small non-coding RNAs of approximately 22 nucleotides in length that function to suppress protein expression predominantly by base pairing with the 3′-untranslated region (3′-UTR) of their target mRNAs [5]. In recent decades, accumulating evidence has suggested that miRNAs may act as tumour suppressors or oncogenes by targeting genes involved in cell proliferation, survival, apoptosis, and metastasis [6, 7]. Furthermore, numerous studies have indicated that miRNAs are involved in the pathogenesis of gliomas [8, 9]. Recent profile studies have shown that microRNA-375 (miR-375) is implicated in various cancers. Specifically, miR-375 downregulation has been reported in gastric cancer, cervical cancer, pancreatic ductal adenocarcinoma, and hepatocellular carcinoma. In these malignancies, miR-375 functions as a tumour-suppressor [10]. However, the role of miR-375 in human glioma remains unclear.

Moreover, exosomal secretion of miR-375 was reported in rectal [11], breast [12], and prostate cancer [13] cells. Exosomes are membrane-derived extracellular vesicles, 40–150 nm in diameter, released by multiple cell types. They are known to contain various cargo materials, including proteins, mRNA, and miRNA [14]. Exosomes play important roles in intercellular communication, serving as carriers for the exchange of various molecular constituents between cells [15–19]. However, little is currently known regarding the pathophysiological role of exosome secretion [20]. Recently, selective packaging of specific miRNAs into exosomes has been reported [15, 21, 22]. In tumours this process can be induced under conditions that promote or inhibit malignancy. For example, some exosome-packaged miRNAs exhibit carcinogenic activity, while others serve as cancer suppressors [23, 24]. Therefore, the exosomal release of selectively packaged miRNAs has been proposed as a means of eliminating tumour suppressors from cancer cells, as is the case with miR-23b-3p and miR-193a in bladder and colon cancer, respectively [24, 25].

In the present study, we found that in glioma cells miR-375 was selectively recruited to exosomes for secretion. This event allowed for the elimination of tumour-suppressive miR-375, thus promoting cancer cell *growth*. The inhibitory effect of miR-375 on gliomas was found to rely on the inactivation of the *connective* *tissue* *growth* *factor* (*CTGF*)-*epidermal* *growth* *factor* *receptor* (EGFR) signalling pathway. In conclusion, the selective exosomal packaging of miR-375 in glioma cells was associated with the stimulation of *CTGF*-EGFR signalling, thus promoting the development of glioma.

## Methods

### Database analysis

miRCancer is a publicly available database (http://mircancer.ecu.edu/) that provides a complete set of miRNA expression profiles in various human cancers. This information is automatically extracted from Pubmed. Additionally, DbDEMC (database of Differentially Expressed MiRNAs in human Cancers) is an integrated database containing high-throughput data for differentially expressed miRNAs in human cancers. DbDEMC 2.0 is also publicly available at http://www.picb.ac.cn/dbDEMC. Gene Expression Omnibus (GEO, http://www.ncbi.nlm.nih.gov/geo/) database is a public functional genomics data repository, which store curated gene expression datasets, original series, and platform records. The CGGA database (publicly available at http://cgga.org.cn/index.jsp) is a user-friendly web application for data storage and analysis of brain tumour datasets from over 2000 samples of Chinese cohorts.

### Cell culture and selection

The human normal glial cell line HEB, as well as the human glioma cell lines U87, U251, A172, LN18, SHG-44, and U138 were purchased from the American Type Culture Collection (ATCC) and authenticated by short tandem repeat DNA profiling. Primary glioma cells, G15, were isolated from tumour tissues of glioma patients, as previously described [26]. G15 cells were derived from grade IV astrocytoma. All cells were cultured in high-glucose Dulbecco’s modified eagle medium (DMEM; Gibco, Grand Island, USA) supplemented with 10% foetal bovine serum (FBS; Gibco, Grand Island, USA), 100 μg/mL streptomycin, and 100 U/mL penicillin. The cells were maintained in a humidified chamber containing 5% CO_2_ at 37 °C. The medium was replaced three to four times per week. Cell passaging was carried out when cell confluence reached approximately 80%. Quantitative real-time polymerase chain reaction (qRT-PCR) was used to detect the expression of miR-375 in the six glioma cell lines, and the two cell lines with the lowest expression of miR-375 were selected for subsequent experiments. Meanwhile, the primary glioma cell line, G15, was also selected for subsequent experiments.

### Preparation of lentiviral vector and transfection

MiR-375 mimic and miR-control were purchased from GenePharma (Shanghai, China). MiRNA transfections were performed using Lipofectamine 2000 (Invitrogen, Carlsbad, USA), according to the manufacturer’s instructions. The extent of overexpression was evaluated by qRT-PCR 24 h after transfection. MiR-375 mimic and miR-control lentiviruses were synthesised by Genechem (Shanghai, China). For stable overexpression of miR-375 in glioma cells, miR-375 mimic or miR-control lentiviruses were added to cells, according to the manufacturer’s protocol. After 24 h, clones with stable expression were selected by incubating cells for 3 weeks with 5 μg/mL puromycin (Sigma, St Louis, USA) in complete medium containing 10% FBS. The sequences of miR-control and miR-375 mimic were 5′-TTCTCCGAACGTGTCACGT-3′ and 5′-TTTGTTCGTTCGGCTCGCGTGA-3′, respectively.

### Treatment of cells with *CTGF* or *epidermalgrowthfactor* (EGF)

After miR-375 was overexpressed in glioma cells, the cells were treated with 200 ng/mL *CTGF* (Peprotech, Rocky Hill, USA) or 20 ng/mL EGF (Abcam, Cambridge, UK) for 24 h.

### Cell counting kit assay

The glioma cells were seeded in 96-well plates (Costar, Cambridge, USA) at a density of 3 × 10^3^ cells/well, and cultured at 37 °C for 3–5 days. Viable cells were analysed with the Cell Counting Kit-8 (CCK-8; Dojindo, Kumamoto, Japan) according to the manufacturer’s guidelines using a microplate reader (BioTek, Winooski, USA) at 450 nm.

### 5-ethynyl-2′-deoxyuridine (EdU) cell proliferation assay

The rate of cell proliferation was measured using an EdU cell proliferation assay kit (KeyGEN BioTECH, Nanjing, China), according to the manufacturer’s protocol. The glioma cells were incubated with 250 μL of EdU solution for 2 h at 37 °C, and then fixed in 4% paraformaldehyde for 15 min, permeabilised with 0.4% Triton X-100 (Sigma, St Louis, USA) for 10 min, and incubated with Apollo®reagent (250 μL) for 30 min. Subsequently, the nuclei were stained with 4′,6-diami-dino-2-phenylindole (DAPI; Sigma, St Louis, USA) for 30 min, and images were obtained using an inverted fluorescence microscope. The proportions of Edu-positive and DAPI-positive cells were then calculated.

### Wound healing assay

At least five transverse lines were drawn on the back of each well of a 6-well plate using a marker pen. Next, 5 × 10^5^ cells were added to each well and incubated overnight. Vertical lines were then drawn using a pipette tip. After removal of the detached cells, serum-free medium was added, and the cells were incubated in culture with 5% CO_2_ at 37 °C. Finally, the cells were photographed at 0, 24, and 48 h.

### Transwell migration and invasion assays

The migration and invasion assays were performed using cell culture inserts with 8 μm pores and 24-well plates (Costar, Cambridge, USA). For the invasion assay, the upper chamber was coated with 50 μL of Matrigel (BD Biosciences, San Jose, USA). To assess migration, the filters were not precoated with Matrigel. The glioma cells were added to the top chamber in serum-free medium. The bottom chamber was filled with 10% FBS DMEM. After 24 or 48 h of incubation, the cells in the top chamber were removed using a cotton swab, and the membrane was fixed in 4% paraformaldehyde for 15 min, and stained with Crystal Violet for 15 min. Images of five random fields were taken for each well, and quantification was performed by using ImageJ (NIH, Bethesda, USA).

### Bioinformatic analysis of miRNA

The TargetScan (http://www.targetscan.org), Pictar (https://pictar.mdc-berlin.de/), miRanda (http://www.microrna.org/microrna/home.do), and StarBase (http://starbase.sysu.edu.cn/index.php) algorithms were used to identify putative targets of miR-375.

### RNA extraction and qRT-PCR

Total RNA from glioma cells was isolated using TRIzol reagent (Invitrogen, Carlsbad, USA). Exosome RNA extraction was conducted using the miRNeasy Mini Kit (Qiagen, Hilden, Germany). The PrimeScriptTMRT reagent kit and the gDNA Eraserkit (TaKaRa, Tokyo, Japan) were used to reverse transcribe 1 μg of total RNA into complementary DNA. An SYBR® Premix Ex TaqTM kit (TaKaRa, Tokyo, Japan) was used for qRT-PCR on a LightCycler 480 instrument (Roche, Indianapolis, USA). The relative RNA expression was determined by the comparative Ct (2^-ΔΔCt^) method. The primers were provided by Sangon Biotech Ltd. Company (Shanghai, China; Table 1).

**Table 1:** qRT-PCR primer sequences

Genes	Primer sequences
miR-375	F: 5′-GCGTTTGTTCGTTCGGCTC-3’
	R: 5′-AGTGCAGGGTCCGAGGTATT-3’
U6	F: 5′-CTCGCTTCGGCAGCACA-3’
	R: 5′-AACGCTTCACGAATTTGCGT-3’
*CTGF*	F: 5′-ATTCTGTGGAGTATGTACCGAC-3’
	R: 5′-GTCTCCGTACATCTTCCTGTAG-3’
GAPDH	F: 5′-GGCACCGTCAAGGCTGAGAAC-3’
	R: 5′-TGCTGATGATCTTGAGGCTGTTGTC-3’

*F* forward primer, *R* reverse primer

### Western blot analysis

Total and exosomal proteins were extracted using the Whole Cell Lysis Assay (KeyGEN BioTECH, Nanjing, China). Protein extracts were separated by 8–12% SDS-PAGE and transferred onto PVDF membranes (Millipore, Billerica, USA). After blocking with 5% BSA (Sigma, St Louis, USA), the membranes were incubated with primary antibodies (Supplementary Table S1) overnight at 4 °C. Subsequently, the membranes were incubated with secondary antibodies conjugated with horseradish peroxidase for 1 h at room temperature. The protein bands were visualised by enhanced chemiluminescence (ECL; Millipore, Bedford, USA), while protein band intensities were analysed by ImageJ software and normalised to GAPDH.

### Enzyme linked immunosorbent assay (ELISA)

The cell culture medium was collected 72 h after treatments. *CTGF* secretion was detected by ELISA (Proteintech, Chicago, USA) according to the manufacturer’s instructions.

### Exosome isolation

The exosomes were removed from FBS by ultracentrifugation at 100,000×*g* for 8 h (Exo-free-FBS). When the confluence of glioma cell lines reached approximately 80%, DMEM with 10% Exo-free-FBS was added to the cells and incubated at 37 °C with 5% CO_2_ for 48 h. The cell medium was then collected and centrifuged at 4 °C and 300×*g* for 10 min. The supernatant was collected and recentrifuged for 15 min at 2000×*g* and 4 °C and for another 15 min at 5000×*g*. The supernatant was then collected and centrifuged at 12,000×g for 30 min. The final supernatant was collected and subjected to ultracentrifugation at 100,000×*g* and 4 °C for 70 min (Beckman Coulter, Brea, USA). The exosomal pellets were washed with sterilised PBS and ultracentrifuged at 100,000×*g* for 1 h. Subsequently, exosomes were resuspended in 100 μL of PBS and stored at − 80 °C until use.

### Exosome characterisation

The morphology of the isolated exosomes was examined by transmission electron microscopy (TEM; Hitachi HT7650, Tokyo, Japan). Briefly, the isolated exosomes were fixed with 2% paraformaldehyde and spotted onto a glow-discharged copper grid on filter paper. Afterwards, the copper grid was dried for 15 min at room temperature. The samples were subsequently stained with 2% uranyl acetate and dried for 10 min. Subsequently, the samples were examined at 100 keV. The size distribution of the isolated exosomes was analysed by a Zetasizer Nano-Zs instrument (Malvern Panalytical, Worcestershire, UK) according to the manufacturer’s instructions. The exosome concentration was evaluated using the bicinchoninic acid (BCA) protein assay kit (KeyGEN BioTECH, Nanjing, China), as per manufacturer’s instructions. For analysis of exosomal protein markers, western blotting assays with anti-CD9, anti-ALIX, anti-TSG101, and anti-calnexin antibodies (Supplementary Table S1) were performed.

### Exosome secretion and uptake assay

GW4869 (10 μM; Sigma, St Louis, USA) and monensin (1 μM; MedChemExpress, Shanghai, China) were used to inhibit and stimulate exosome release, respectively. To monitor exosomal trafficking, exosomes were labelled with a PKH26 fluorescent cell linker kit (Sigma, St Louis, USA). PKH26-labelled exosomes were washed in PBS and centrifuged at 100,000×*g* for 20 min at 4 °C, collected, and resuspended in PBS. PKH26-labelled exosomes were then incubated with glioma cells for 24 h. Finally, the uptake of exosomes by glioma cells was assessed by confocal fluorescence microscopy and flow cytometry.

### Serum samples from patients with glioma

All blood samples were collected at the Department of Neurosurgery, Zhujiang Hospital, Southern Medical University of China. The enrolled subjects included 25 males and 16 females, with an average age of 44 years (range, 5–73). Nine of the 31 gliomas were classified as low-grade (WHO I-II), and 22 were classified as high-grade (WHO III-IV). The clinicopathological features of patients are shown in Table 2. Moreover, blood samples from ten healthy humans were extracted as controls. All blood samples were centrifuged at 3000×*g* for 10 min at 4 °C for plasma extraction. Informed consent was obtained from all patients. Both the study protocol and the informed consent were approved by the Ethical Committee of Zhujiang Hospital.

**Table 2:** Analysis of clinical parameters related to the expression of miR-375 in plasma exosomes of glioma

Clinicopathological parameters	miR-375 expression		*p* value
	High (*n* = 20)	Low (*n* = 21)	
Gender			0.606
Male	13	12	
Famale	7	9	
Age (years)			0.087
< 50	14	7	
> 50	8	12	
Glioma grade			**0.009**
0	1	9	
1–2	4	5	
3–4	15	7	

**High / Low** indicate above and below the median expression level (median = 7.3). 0.003 represent significant *p*-value

### Enrichment of plasma an astrocyte-derived exosomes (ADEs) for RNA extraction

For total exosome purification from plasma, 2 mL of plasma were obtained from 5 mL of blood and diluted with 10 mL of PBS. Next, the same procedure used for exosome purification from cultured cells was followed. To enrich ADEs, total exosomes were resuspended in 0.35 mL of PBS and incubated for 60 min at room temperature with 1.5 μL of mouse anti-human glutamine aspartate transporter (GLAST) biotinylated antibody (Miltenyi Biotec, Auburn, USA) in 50 μL of 3% BSA per tube with mixing, followed by addition of 10 μL of streptavidin-agarose UltraLink resin (Thermo Fisher Scientific, Waltham, USA) in 40 μL of 3% BSA, and incubated for 30 min at room temperature with mixing. After centrifugation at 800×*g* for 10 min at 4 °C and removal of the supernatant, each pellet was resuspended in 100 μL of cold 0.05 M glycine-HCl (pH 3.0) by gentle mixing for 10 s and centrifuged at 4000×*g* for 10 min, all at 4 °C. Supernatants were then transferred to clean tubes containing 25 μL of 10% BSA and 10 μL of 1 M Tris-HCl (pH 8.0) and mixed before addition of 700 μL QIAzol Lysis Regent. Subsequently, ADEs RNA extraction was conducted using the miRNeasy Mini Kit (Qiagen, Hilden, Germany).

### Intracranial xenograft assay in nude mice

First, to explore the role of miR-375 in vivo, U87 luciferase cells (U87-Luc; 1 × 10^5^) were transfected with miR-375-mimic (U87-Luc-mimic) or miR-control (U87-Luc-control) lentiviruses, and stereotactically implanted into the brains of 4-week-old nude mice. Each group included eight mice (Central Animal Facility of Southern Medical University, Guangzhou, China). Second, to examine the properties of exosomes in vivo, U87-Luc-mimic cells were stereotactically implanted into the brains of 32 nude mice. On day 2 post-tumour implantation, 16 mice were randomly selected and subjected to three weekly intraperitoneal injections of GW4869 (1.25 μg/g), while the other 16 mice received three weekly injections of DMSO (the GW4869 solvent). Bioluminescent imaging (IVIS Lumina II, Caliper, USA) was used to monitor intracranial tumour *growth*, and the survival of the animals was recorded every day. After 3 weeks, eight mice per group were sacrificed. The brains were collected and stained with hematoxylin and eosin (H&E) and immunohistochemical (IHC). The remaining nude mice were used to generate survival curves. Meanwhile, we repeated the animal experiment with the primary glioma cell G15. The protocols used in this study were approved by the Animal Care and Use Committee of Southern Medical University.

### Immunohistochemistry (IHC)

Paraffin-embedded *tissue* sections (4 μm in thickness) were subjected to IHC assays. Briefly, after deparaffinisation, rehydration, antigen retrieval, and blocking of endogenous peroxidase activity, the sections were exposed to specific antibodies against *CTGF*, Ki-67, and MMP9, (Supplementary Table S1) and subsequently incubated with appropriate secondary antibodies. Next, the signals were developed using 3,3′-diaminobenzidine (DAB; Sigma, St Louis, USA) solution, followed by counterstaining with haematoxylin (Sigma, St Louis, USA). The staining intensity was measured by ImageJ software in five random regions, and the average values were used to obtain the protein expression levels.

### Statistical analysis

All statistical analyses were performed using GraphPad Prism 6 (GraphPad Software Inc., La Jolla, USA). The data were expressed as mean ± standard error of three independent experiments. The statistical significance was assessed by Student’s *t*-test or one-way analysis of variance (ANOVA) with Bonferroni correction for multiple comparisons. The survival rates were obtained using the Kaplan–Meier method, and the differences in mortality were evaluated by log-rank-test. All tests were two-sided, and *P*-values < 0.05 were set as the threshold for statistical significance.

## Results

### MiR-375 is poorly expressed in glioma

**Fig. 1:**
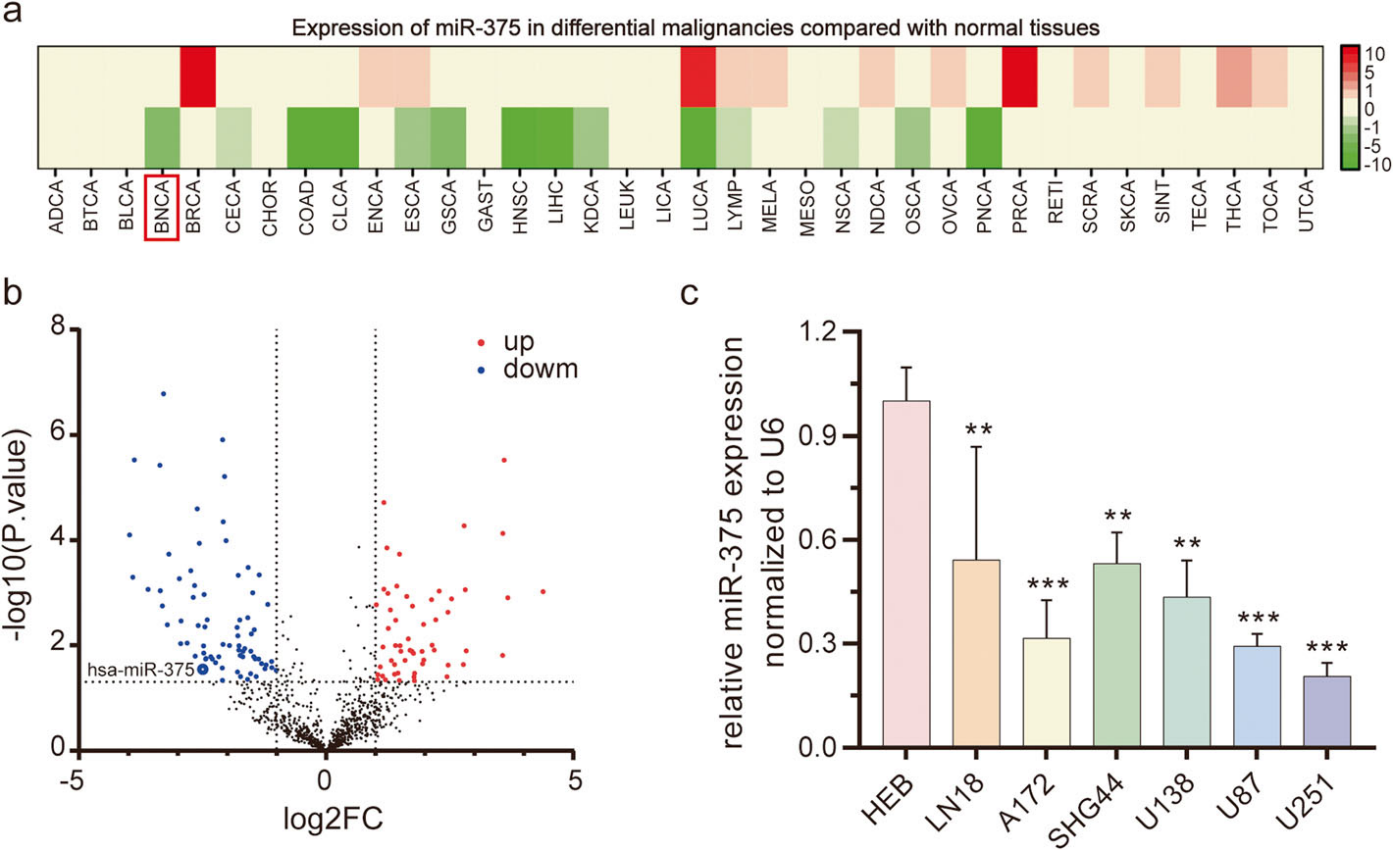
MiR-375 is under-expressed in glioma *tissue* and glioma cell lines. **a.** Calculation of miR-375 expression through dbDEMC2 software. MiR-375 expression in multiple human maligancies, including brain cancer (BNCA). **b.** Volcano plots of the distributions of miR-375. **c.** qRT-PCR assay depicting expression of miR-375 in glioma cell lines compared to HEB cells. All experiments were repeated independently three times. Data are presented as mean ± standard deviation. **p < 0.01; ***p < 0.001

To explore the expression of miR-375 in glioma, the miRCancer database (http://mircancer.ecu.edu) was searched. Two studies were found to report decreased expression of miR-375 in gliomas [27, 28]. In addition, miR-375 exhibited a relatively low expression in multiple human malignancies, including brain cancer (BNCA), as assessed by dbDEMC2 analysis (http://www.picb.ac.cn/dbDEMC; Fig. 1a). Next, miR-375 expression was investigated in 82 patients with glioma from the GEO database (http://www.ncbi.nlm.nih.gov/geo/; GSE25632). In line with the results obtained with dbDEMC2, GEO analysis revealed the low expression of miR-375 in glioma. (Fig. 1b). We found that miR-375 was downregulated in human glioma tissues. To confirm this result, we used qRT-PCR to analyse miR-375 expression in six glioma cell lines and normal colloidal cells. MiR-375 was poorly expressed in all glioma cell lines compared to normal colloidal cells (Fig. 1c). MiR-375 was lowest in the U87/U251 glioma cells, which were, therefore, selected for subsequent experiments. Meanwhile, similar results were obtained in the primary glioma cell, G15 (Fig. S2a), therefore, G15 was also selected for subsequent experiments.

### MiR-375 overexpression inhibits glioma cell proliferation, migration, and invasion in vitro and in vivo

**Fig. 2:**
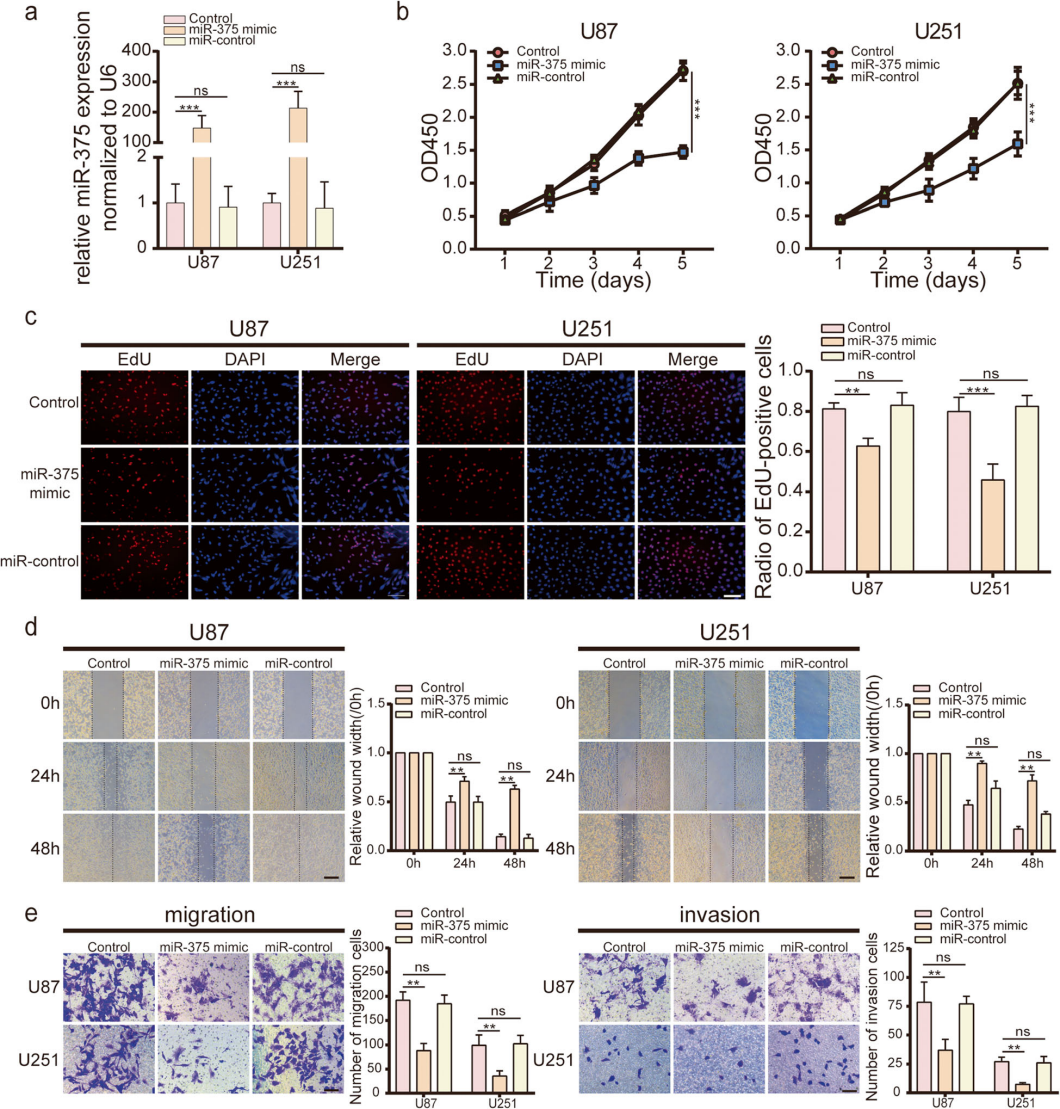
MiR-375 regulates cell viability, cell migration and cell invasion in glioma cell lines. **a**. MiR-375 overexpression in U87 and U251 cells were validated by qRT-PCR. **b, c**. CCK-8 and EdU assays show that miR-375 upregulation inhibits cell proliferation in both U87 and U251 cells. Scale bar = 100 μm. **d**. Cell migration was determined by a wound healing assay. The migration distance was measured at 0, 24, and 48 h after the cells were scratched. Scale bar, 400 μm. **e**. The effect of miR-375 overexpression on the migration (left) and invasion (right) of both U87 and U251 cells, as examined using a transwell assay. Scale bar = 100 μm. All experiments were repeated independently three times. Data are presented as mean ± standard deviation. **p < 0.01; ***p < 0.001. ns, not significant

To explore the role of miR-375 in glioma cells, we transfected U87, U251, and G15 cells with a lentiviral plasmid containing pre-miR-375. The expression of mature miR-375 in stable transfectants was confirmed by qRT-PCR (Fig. 2a and Fig. S2b). CCK-8 and EdU assay results revealed that miR-375 overexpression significantly reduced the proliferation rate of U87/U251 cells, as compared to control cells (Fig. 2b-c). To further confirm these results, we repeated the CCK-8 experiment with G15 cells. Compared with the control group, miR-375 also inhibited the proliferation of G15 cells (Fig. S2e). In addition, the function of miR-375 was examined in vivo. To this end, tumour formation was induced in nude mice by intracranial injection of U87-Luc-mimic cells or U87-Luc-control cells (Fig. S1a). Consistently with the in vitro results, miR-375 inhibited tumour formation in nude mice (Fig. S1b-c). To visualise the proliferating cells, immunohistochemical staining for Ki-67 was performed. As expected, lower Ki-67 expression was observed in U87-Luc-mimic-derived tumours compared to U87-Luc-control-derived tumours (Fig. S1d). Next, we explored whether miR-375 influences the migration and invasion of glioma cells. A wound healing assay showed that miR-375 significantly inhibited the migration of U87/U251 glioma cells (Fig. 2d). A transwell assay was then performed to determine the impact of miR-375 on glioma cell mobility. MiR-375 overexpression inhibited the migration and invasion of U87, U251, and G15 cells (Fig. 2e and Fig. S2f). In addition, xenograft tumours derived from U87-Luc-mimic cells exhibited reduced invasion ability compared to U87-Luc-control-derived tumours, in line with the above-described in vitro results (Fig. S1e). Hence, restoration of miR-375 expression suppressed the proliferation, migration, and invasion of glioma cells.

### *CTGF* is a direct target of miR-375

**Fig. 3:**
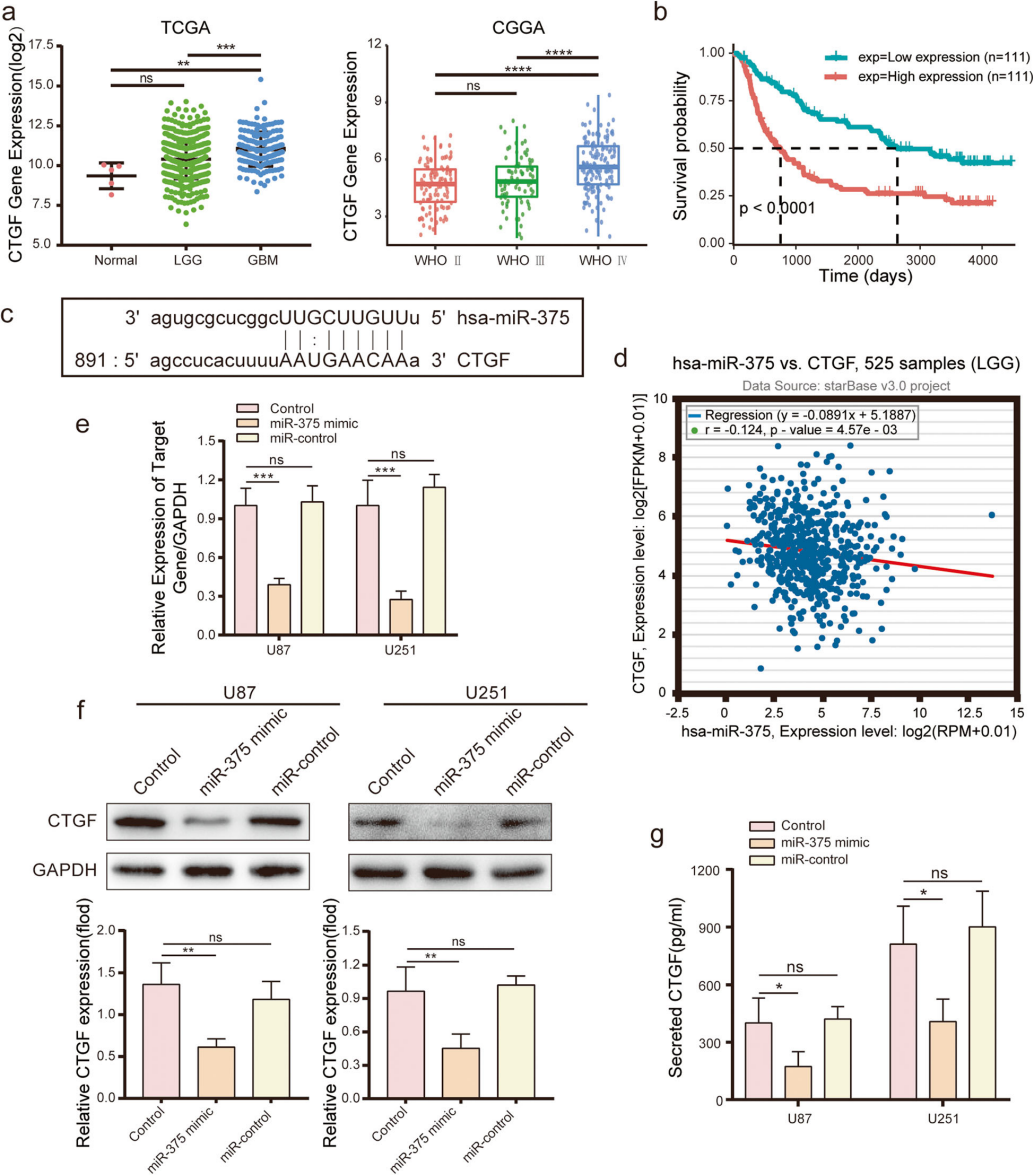
*CTGF* is a direct target of miR-375. **a.** Correlation between *CTGF* expression level and glioma grade. *CTGF* expression levels in glioma in TCGA RNA-seq (left), CGGA RNA-seq (right) databases. **b.** Prognosis efficiency of *CTGF* in WHO grade patients within the CGGA RNA-seq data. **c.** Predicted binding sequence of human hsa-miR-375 and its binding site in the 3′-UTR of *CTGF* presented for alignment. **d.** StarBase v2.0 analysis demonstrating a negative correlation between miR-375 and *CTGF* in low-grade glioma (LGG). **e.** Expression of candidate target gene *CTGF* in U87 and U251 cells as assessed by qRT-PCR following overexpression of miR-375. **f.** Western blot analysis of *CTGF* in U87 (left) and U251 (right) cells following miR-375 treatment. GAPDH was used as an internal control. **g.** *CTGF* levels measured by ELISA following miR-375 treatment. All experiments repeated independently three times. Data are presented as mean ± standard deviation. *p < 0.05; **p < 0.01; ***p < 0.001. ns, not significant

A PubMed literature search for potential targets of miR-375 identified *CTGF* as a candidate miR-375 target in colon cancer [29]. Notably, *CTGF* has been implicated in glioma progression [30]. To further explore this observation, the level of *CTGF* expression was analysed in glioma tissues from the CGGA and TCGA databases. In contrast to miR-375, *CTGF* protein level was upregulated in gliomas (Fig. 3a). Next, the prognostic value of *CTGF* was addressed by examining the overall survival rate of glioma patients from the CGGA database. Notably, patients with high *CTGF* expression displayed a significantly poorer prognosis compared to those with low *CTGF* expression (Fig. 3b). Therefore, to determine whether *CTGF* could serve as a target for miR-375 in glioma cells, four public miRNA databases (TargetScan, Pictar, miRanda, and StarBase) were searched for potential miR-375 targets involved in tumour progression. The analysis of all four databases identified *CTGF* as a candidate target for miR-375 (Fig. 3c). Furthermore, the StarBase database revealed that the miR-375 level was inversely correlated with *CTGF* expression in low-grade glioma (Fig. 3d). To address the relationship more directly between miR-375 and *CTGF* in glioma cells, a mature miR-375 mimic was transfected into U87, U251, and G15 cells. MiR-375 overexpression significantly downregulated *CTGF* expression at both the mRNA and protein levels (Fig. 3e-f and Fig. S2c-d). In addition, ELISA assays showed that miR-375 overexpression was associated with reduced *CTGF* secretion (Fig. 3g). Notably, the interaction between the 3′-UTR of *CTGF* mRNA and miR-375 is well-documented [29, 31].

### MiR-375 regulates the *CTGF*-EGFR signalling pathway

**Fig. 4:**
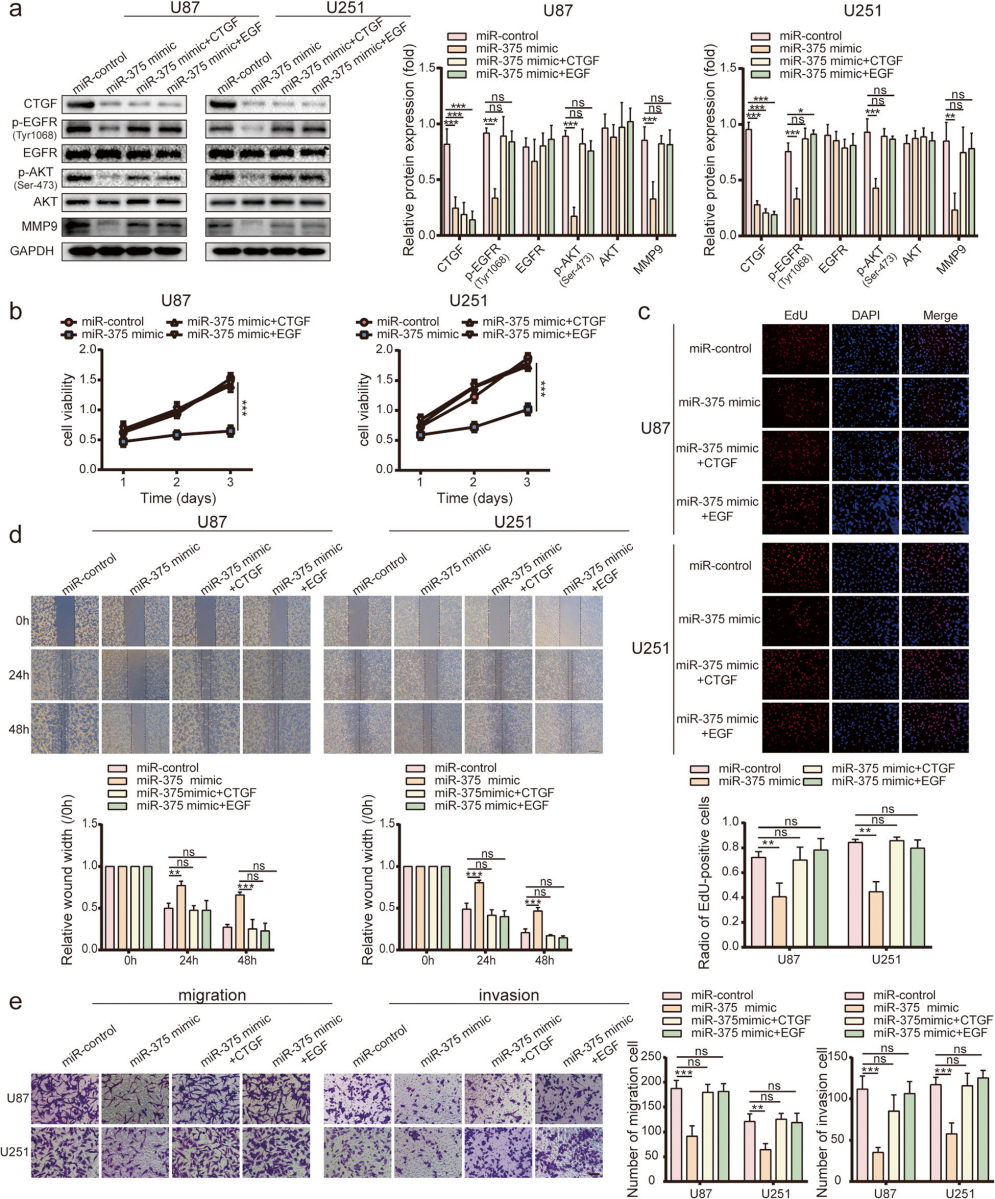
miR-375 regulates the proliferation and invasion of glioma through the *CTGF*-EGFR signalling pathway. **a.** Western blot analysis of *CTGF*, p-EGFR (Tyr1068), EGFR, p-AKT (Ser-473), AKT, and MMP9 in U87 and U251 cells. MiR-375 significantly down-regulates *CTGF* and its downstream molecules p-EGFR (Tyr1068), p-AKT (Ser-473), and MMP9. *CTGF* or EGF reversed the protein levels of p-EGFR (Tyr1068), p-AKT (Ser-473), and MMP9. **b, c.** Effect of *CTGF* or EGF on the *growth* inhibitory effect of miR-375, as determined by CCK-8 and EdU analysis. Scale bar = 100 μm. **d.** Wound healing analysis demonstrating the effect of *CTGF* or EGF on the inhibitory effect of miR-375 on glioma migration. Scale bar = 400 μm. **e.** Effect of *CTGF* or EGF addition on the inhibitory effect of miR-375 on glioma cell migration (left) and invasion (right) using Transwell analysis. Scale bar = 100 μm. All experiments were repeated independently three times. Data are presented as mean ± standard deviation. *p < 0.05; **p < 0.01; ***p < 0.001. ns, not significant

*CTGF* is a matricellular protein that binds to the EGFR and regulates tumour progression. To determine whether miR-375 suppresses *CTGF*-EGFR signalling, pre-miR-375 was transfected into glioma cell lines, and the levels of proteins associated with *CTGF*-EGFR signalling were analysed by western blot. Overexpression of miR-375 in U87, U251, and G15 cells was associated with significant downregulation of *CTGF*, as well as of its downstream effectors p-EGFR (Tyr1068), p-AKT (Ser-473), and MMP9 (Fig. 4a and Fig. S2d). To directly verify the role of miR-375 in the regulation of the *CTGF*-EGFR pathway in gliomas, the impact of *CTGF* or EGF-induced EGFR stimulation on the expression of proteins involved in *CTGF*-EGFR signalling was evaluated by western blot in miR-375-overexpressing U87, U251, and G15 cells. Interestingly, *CTGF* or EGF clearly prevented the miR-375-induced downregulation of *CTGF*-EGFR signalling-related proteins (Fig. 4a and Fig. S2d). These results indicate that miR-375 inhibits the *CTGF*-EGFR pathway in glioma cells.

### MiR-375 regulates glioma proliferation, migration, and invasion through the *CTGF*-EGFR signalling pathway

Once the role of miR-375 was demonstrated in the *CTGF*-EGFR pathway, we verified whether *CTGF* or EGFR activation could reverse the tumour-suppressive changes induced by miR-375. To this end, U87, U251, and G15 cells were treated with *CTGF* or EGF after transfection with miR-375. The *growth*-suppressive effect of miR-375 was abrogated by *CTGF* or EGF (Fig. 4b-c and Fig. S2e), indicating that these factors were involved in miR-375-induced suppression of cell *growth*. Moreover, cell exposure to *CTGF* or EGF significantly restored cell migration and invasion (Fig. 4d-e and Fig. S2f). Taken together, these findings indicate that miR-375 regulates the proliferation, migration, and invasion of glioma through the *CTGF*-EGFR signalling pathway.

### Exosomes released by glioma cells contain a high level of miR-375

**Fig. 5:**
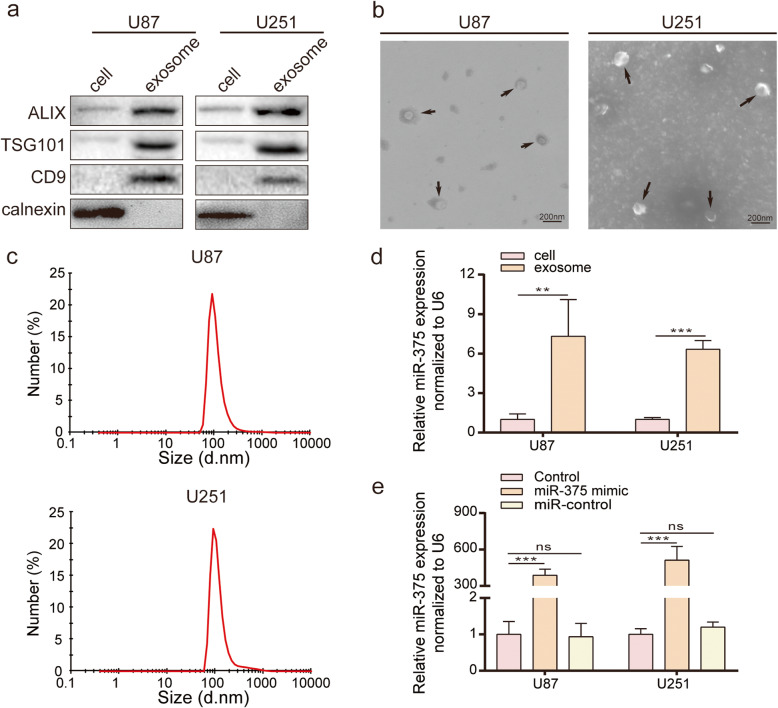
Glioma cell-derived exosomes carry a high expression of miR-375. **a.** Immunoblotting for exosomal markers ALIX, TSG101 and CD9, as well as negative control, calnexin. **b.** Uranyl acetate negative stained TEM images, of exosomes isolated from U87 (left) and U251 cell lines (right). Scale bar = 200 nm. **c.** Size distribution of the isolated exosomes was analysed by Zetasizer Nano-Zs. **d.** Detection of miR-375 relative expression levels in U87 and U251 cells and their derived exosomes by qRT-PCR. **e.** Detection of miR-375 relative expression levels in miR-375 overexpressing U87 and U251 cells derived exosomes by qRT-PCR. All experiments were repeated independently three times. Data are presented as mean ± standard deviation. **p < 0.01; ***p < 0.001. ns, not significant

Selective exosomal packaging and release of miRNAs has been reported to occur in cancer cells as a means of eliminating tumour suppressors [24, 25]. To investigate the occurrence of this phenomenon in gliomas, the presence of miR-375 in the exosomes released by glioma cells was verified. First, the exosomes were collected from U87, U251, and G15 cell culture medium by ultracentrifugation, and the expression of the known exosomal markers, ALIX, TSG101, and CD9, as well as of the endoplasmic reticulum protein, calnexin, was compared by western blot between exosome extracts and donor cells. ALIX, TSG101, and CD9 were enriched in the exosome fractions as compared to the corresponding donor cells, while calnexin was exclusively expressed in donor cells (Fig. 5a and Fig. S3a). The morphology of U87, U251, and G15 exosomes was then analysed by TEM, which revealed that they were round and oval in shape, with a diameter of 40 to 150 nm (Fig. 5b and Fig. S3b). In addition, exosome size was examined using a Zetasizer Nano-Zs analyser, showing that the diameter of most particles was within the typical exosomal range (40–150 nm; Fig. 5c and Fig. S3c). Thus, we concluded that the described procedure led to the isolation of bona fide exosomes from U87, U251, and G15 cells. qRT-PCR was subsequently used to analyse miR-375 expression in U87, U251, and G15 cell exosomes. MiR-375 was significantly enriched in purified exosomes with respect to donor cells (Fig. 5d and Fig. S3d). Moreover, the exosomes of miR-375-overexpressing glioma cells contained a significantly higher level of miR-375 compared to those of control glioma cells (Fig. 5e). These data demonstrate that miR-375 is enriched in glioma cell-derived exosomes.

### The suppression of exosomal miR-375 release leads to increased miR-375 activity in glioma cells

**Fig. 6:**
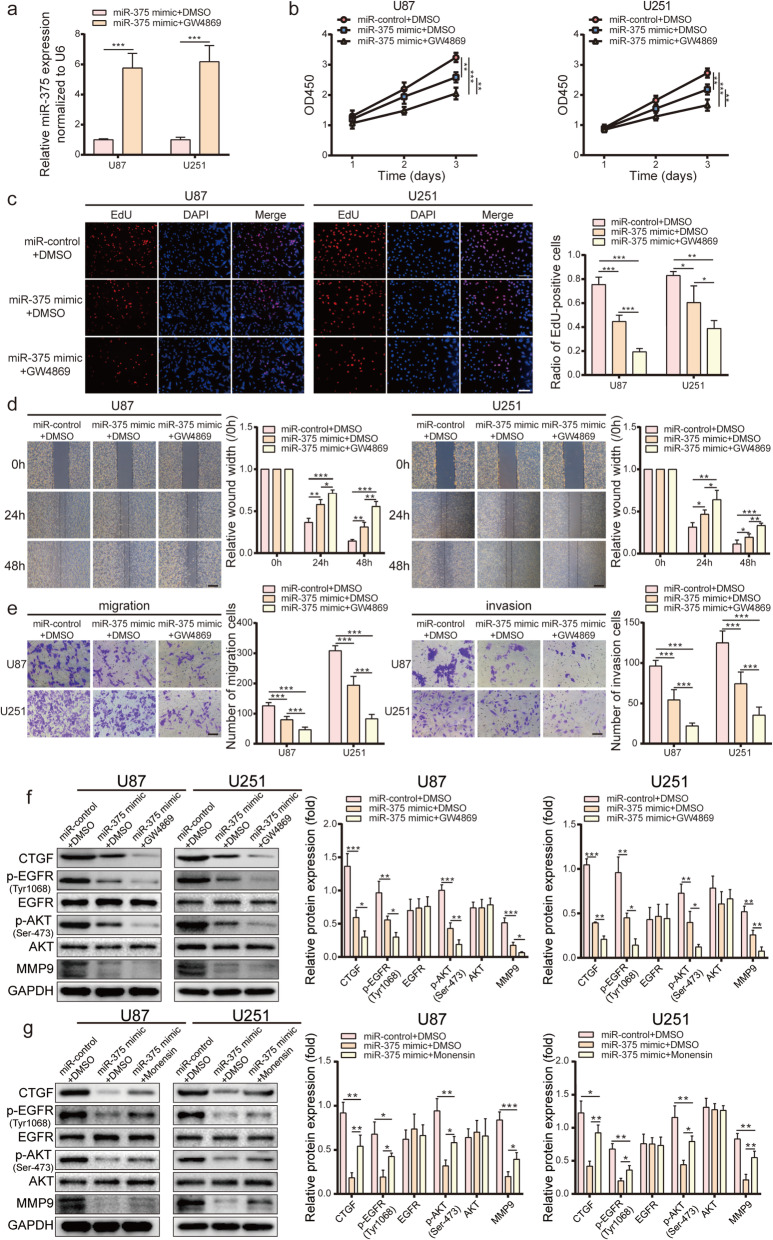
Exosomes regulate the proliferation and invasion of glioma through miR-375-*CTGF*-EGFR signalling pathway. **a.** Changes in miR-375 levels in miR-375 overexpressing U87 and U251 cells treated with or without GW4869 were examined by qRT-PCR. **b-c.** CCK-8 and EdU analysis of the effect elicited by GW4869 on the inhibitory effect of miR-375 on the proliferation of U87 and U251 cells. Scale bar = 100 μm. **d.** Wound healing analysis demonstrating the effect of GW4869 on the inhibitory effect of miR-375 on the migration of U87 and U251 cells. Scale bar = 400 μm. **e.** Transwell analysis showing the effect of GW4869 on the inhibitory effect of miR-375 on glioma cell migration (left) and invasion (right). Scale bar = 100 μm. **f.** Western blot analysis of *CTGF*, p-EGFR (Tyr1068), EGFR, p-AKT (Ser-473), AKT, and MMP9 in U87 and U251 cells. **g.** Effect of monensin on the inhibitory effect of miR-375 on *CTGF* and its downstream molecules p-EGFR (Tyr1068), p-AKT (Ser-473), and MMP9. All experiments were repeated independently three times. Data are presented as mean ± standard deviation. *p < 0.05; **p < 0.01; ***p < 0.001. ns, not significant

To explore whether the suppression of exosomal secretion impacts intracellular miR-375 expression and activity, exosome release by miR-375-overexpressing U87, U251, and G15 cells was blocked by GW4869, an inhibitor of neutral sphingomyelinase 2 (nSMase2). This enzyme converts sphingomyelin to ceramide and has been reported to mediate exosome secretion in neurons, astrocytes, and microglial cells [32, 33]. NSMase2 inhibition reduced exosome secretion in U87, U251, and G15 cells (Fig. S4a-b). Under these conditions, the intracellular level of miR-375 was consistently increased by > 5-fold (Fig. 6a and Fig. S5a). To explore the impact of intracellular miR-375 upregulation on the activity of glioma cells, miR-375-overexpressing U87/U251 cells were treated with GW4869 for 24 h, and CCK-8 and EdU assays were performed to detect cell proliferation. Moreover, wound healing and transwell assays were conducted to evaluate cell migration and invasion. We found that nSMase2 inhibition by GW4869 further suppressed cell proliferation and invasion compared to untreated miR-375-overexpressing cells (Fig. 6b-e). To further confirm these results, we repeated the CCK-8 and transwell experiment with G15 cells. GW4869 was found to further suppress G15 cell proliferation and invasion compared to untreated miR-375-overexpressing cells (Fig. S5b-c). These results indicate that the inhibition of miR-375 exocytosis exacerbates the effects of miR-375 in glioma cells.

### Exosomal miR-375 secretion influences activity of the *CTGF*-EGFR pathway

To verify the relationship between exosomal transport and the miR-375-*CTGF*-EGFR axis, the cells were treated with GW4869 or with the ionophore, monensin, to inhibit and promote exosome release, respectively [34]. A 24 h treatment of miR-375-overexpressing U87, U251, and G15 cells with GW4869 further reduced the expression of *CTGF* and its downstream molecules, p-EGFR (Tyr1068), p-AKT (Ser-473), and MMP9 compared to untreated miR-375-overexpressing cells, as determined by western blotting (Fig. 6f and Fig. S5d). On the other hand, a 24 h treatment with monensin increased exosome release (Fig. S2c-d), and partially restored the protein levels of *CTGF*, p-EGFR (Tyr1068), p-AKT (Ser-473), and MMP9 (Fig. 6g). These results indicated that exosome-mediated miR-375 release impacts the activation status of the *CTGF*-EGFR pathway.

### Exosome-released miR-375 is not efficiently taken back up by glioma cells

**Fig. 7:**
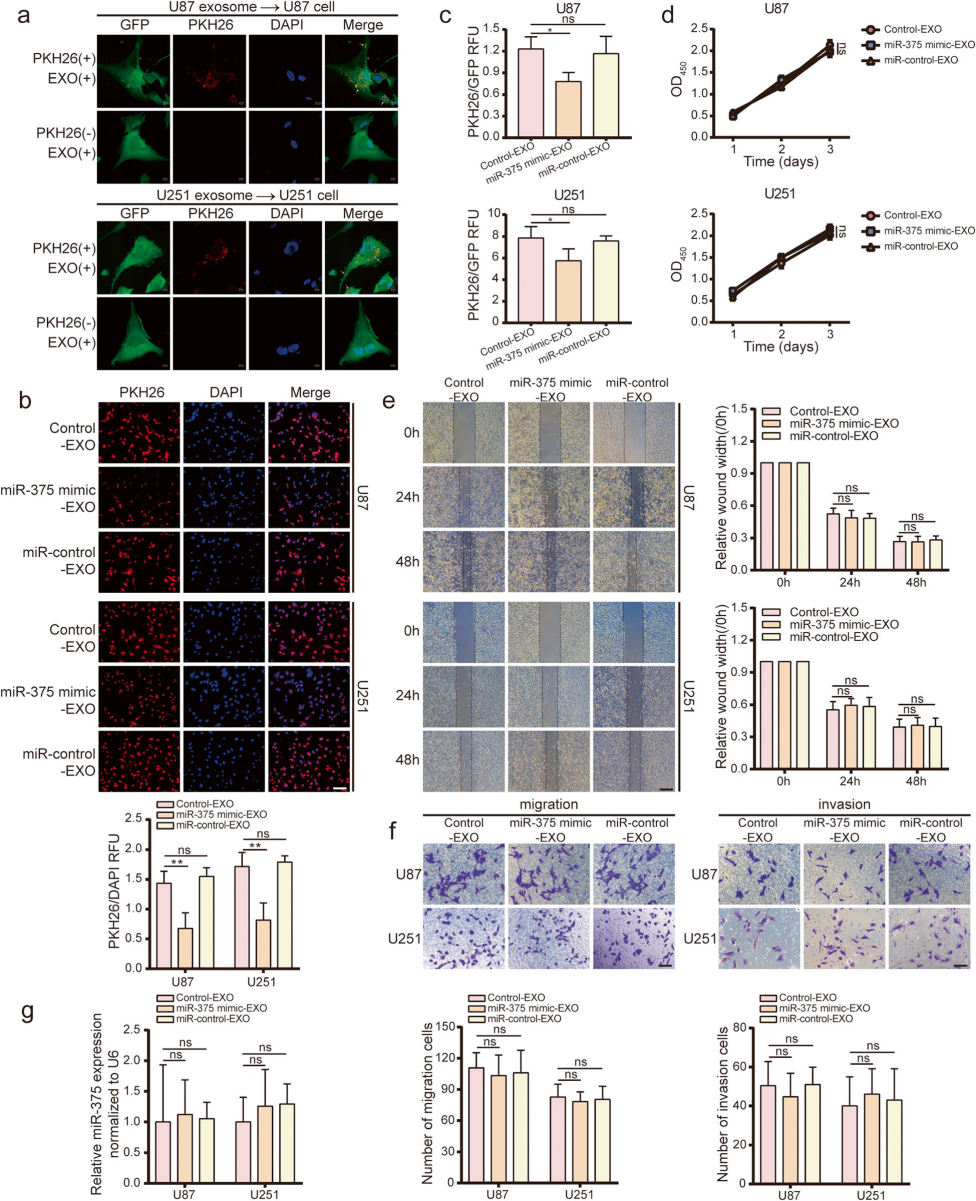
Exosome miR-375 secreted by glioma cells is less efficiently taken up by glioma cells. **a.** Exosomes labelled with PKH26 and incubated with U87/U251 cells for 24 h. Representative laser confocal images showing exosomes merged with U87 (upper panel) and U251 (lower panel). Exosomes are labelled with PKH26 (red), and glioma cells are labelled with GFP (green). Nuclei stained with DAPI (blue). Scale bar = 10 μm. **b.** Fluorescence images of miR-375 mimic-EXO incubated with glioma cells for 24 h. Exosomes are labelled with PKH26 (red). Nuclei stained with DAPI (blue). Scale bar = 100 μm. **c.** The uptake of exosomes by glioma cells was assessed by flow cytometry. **d.** CCK-8 analysis showing the effect of miR-375 mimic-EXO on the proliferation of U87 and U251 cells. **e.** Wound healing analysis demonstrating the effect of miR-375 mimic-EXO on the migration of U87 and U251 cells. Scale bar = 400 μm. **f.** Transwell analysis demonstrating the effect of miR-375 mimic-EXO on glioma cell migration (left) and invasion (right). Scale bar = 100 μm. **g.** qRT-PCR analysis of the effect of miR-375 mimic-EXO on the miR-375 content in U87 and U251 cells. All experiments were repeated independently three times. Data are presented as mean ± standard deviation. *p < 0.05; **p < 0.01. ns, not significant

We next sought to verify whether glioma cells could re-import miR-375 that had been previously excreted via exosomes as waste material. First, the ability of U87/U251 cells to internalise U87/U251 cell-derived exosomes was examined by labelling the exosomes with the red fluorescent dye, PKH26, followed by fluorescence analysis with a laser confocal microscope. Exosomes were internalised by U87/U251 cells and distributed around the nucleus (Fig. 7a). To determine whether miR-375-containing exosomes previously secreted by glioma cells would be imported by glioma cells, exosomes from U87/U251 (control-EXO), U87/U251-miR-375 mimic (miR-375 mimic-EXO), and U87/U251-miR-control (miR-control-EXO) cells were isolated, labelled with PKH26, and incubated with U87/U251 cells for 24 h. Exosome uptake was then evaluated by fluorescence microscopy and flow cytometry. Interestingly, the cellular uptake of miR-375 mimic-EXO was significantly reduced compared to that of miR-control-EXO (Fig. 7b-c). Therefore, although glioma cells were capable of internalising exosomes, miR-375-containing exosomes were poorly imported. To verify whether miR-375-containing exosomes exerted an inhibitory effect in glioma cells, control-EXO, miR-375 mimic-EXO, and miR-control-EXO were incubated with U87/U251 cells for 24 h, after which cell proliferation, migration, and invasion were analysed. No significant differences in any of these processes were observed between the three experimental groups (Fig. 7d-f). Notably, the expression of miR-375 was comparable in U87/U251 cells incubated with control-EXO, miR-375 mimic-EXO, and miR-control-EXO, as assessed by qRT-PCR (Fig. 7g). In summary, miR-375-containing exosomes released by glioma cells are poorly imported by the same cell type.

### Circulating exosomes of patients with glioma contain high levels of miR-375

**Fig. 8:**
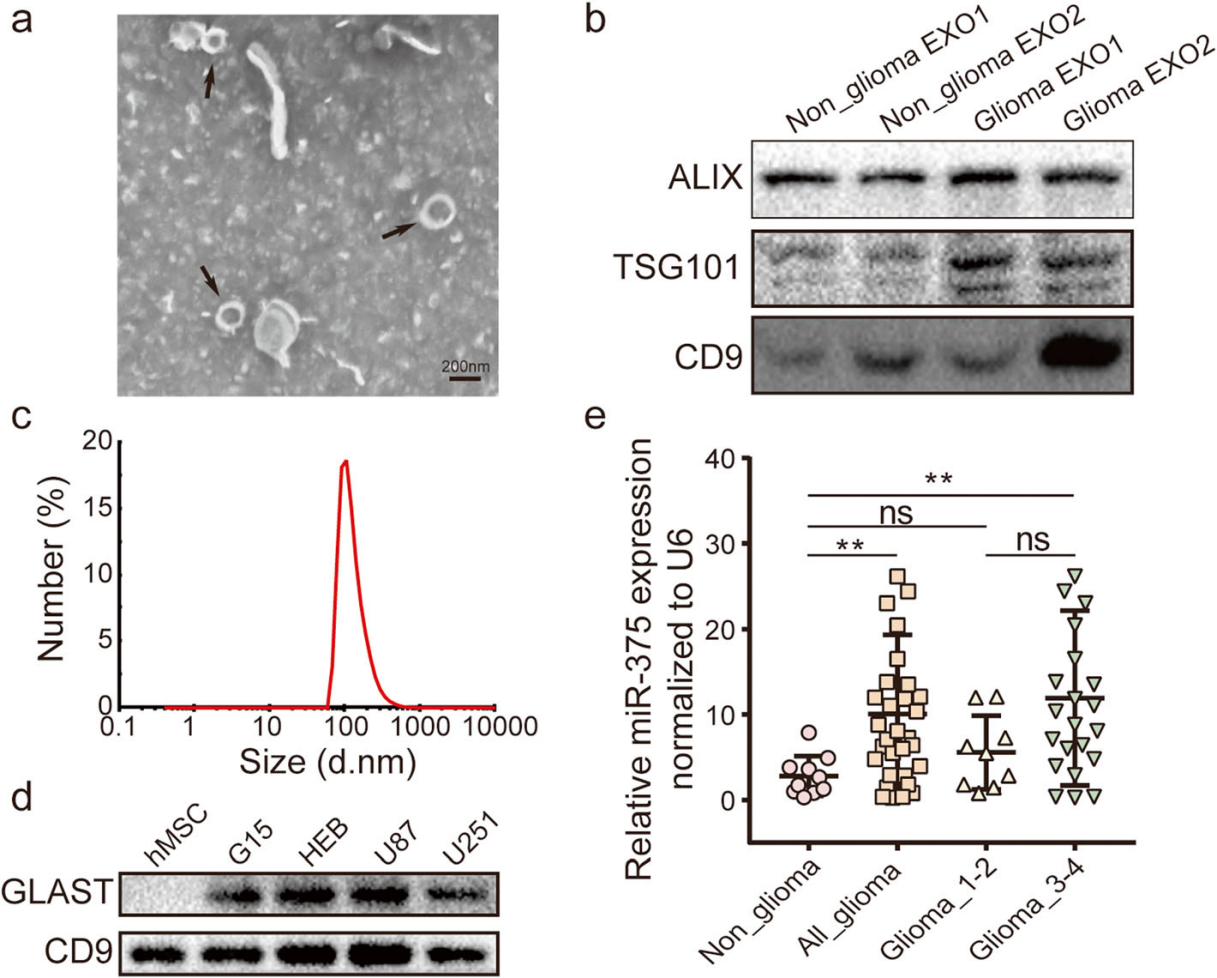
ADEs miR-375 expression in plasma from glioma patients. **a.** Identification of plasma exosomes by TEM. **b.** Detection of ALIX, TSG101 and CD9 protein expression by western blot. **c.** Size distribution of plasma exosome diameter. **d.** Detection of GLAST and CD9 protein expression by western blot. **e.** miR-375 expression in ADEs in plasma from glioma patients and from healthy human donors detected by qRT-PCR. Experimental data presented as mean ± standard deviation. *p < 0.05; **p < 0.01. ns, not significant

To investigate the clinical significance of exosomal miR-375 release, exosomes were isolated from the plasma of glioma patients and analysed by TEM. Exosomes displayed a teacup-like double-sided structure of 40–150 nm (Fig. 8a). In addition, the endosomal markers ALIX, TSG101, and CD9 were examined and analysis of plasma exosomes with a Zetasizer Nano-Zs instrument (Fig. 8b) revealed a size distribution between 40 and 150 nm (Fig. 8c). It has been reported that human marrow stromal cells (hMSC) can also secrete miR-375-containing exosomes [35]. Hence, to avoid interference by hMSC-derived exosomes, we applied the techniques described by Winston et al., and purified plasma exosomes using anti-human GLAST biotinylated antibody [36], since GLAST protein is only expressed on ADEs (Fig. 8d). Next, qRT-PCR was used to determine the level of miR-375 expression in ADE, which was observed to be relatively high in the plasma of patients with high-grade glioma, whereas it did not significantly differ between patients with low-grade glioma and non-glioma volunteers, in line with the above-described cellular experiments (Fig. 8e). Meanwhile, no significant correlation was observed between miR-375 expression and clinicopathological parameters, such as gender or age at diagnosis (Table 2). Taken together, these results indicate that circulating exosomal miR-375 is significantly upregulated in patients with glioma, and that the extent of this upregulation correlates with tumour grade.

### Suppression of exosome secretion enhances miR-375-induced inhibition of glioma proliferation and invasion in vivo

**Fig. 9:**
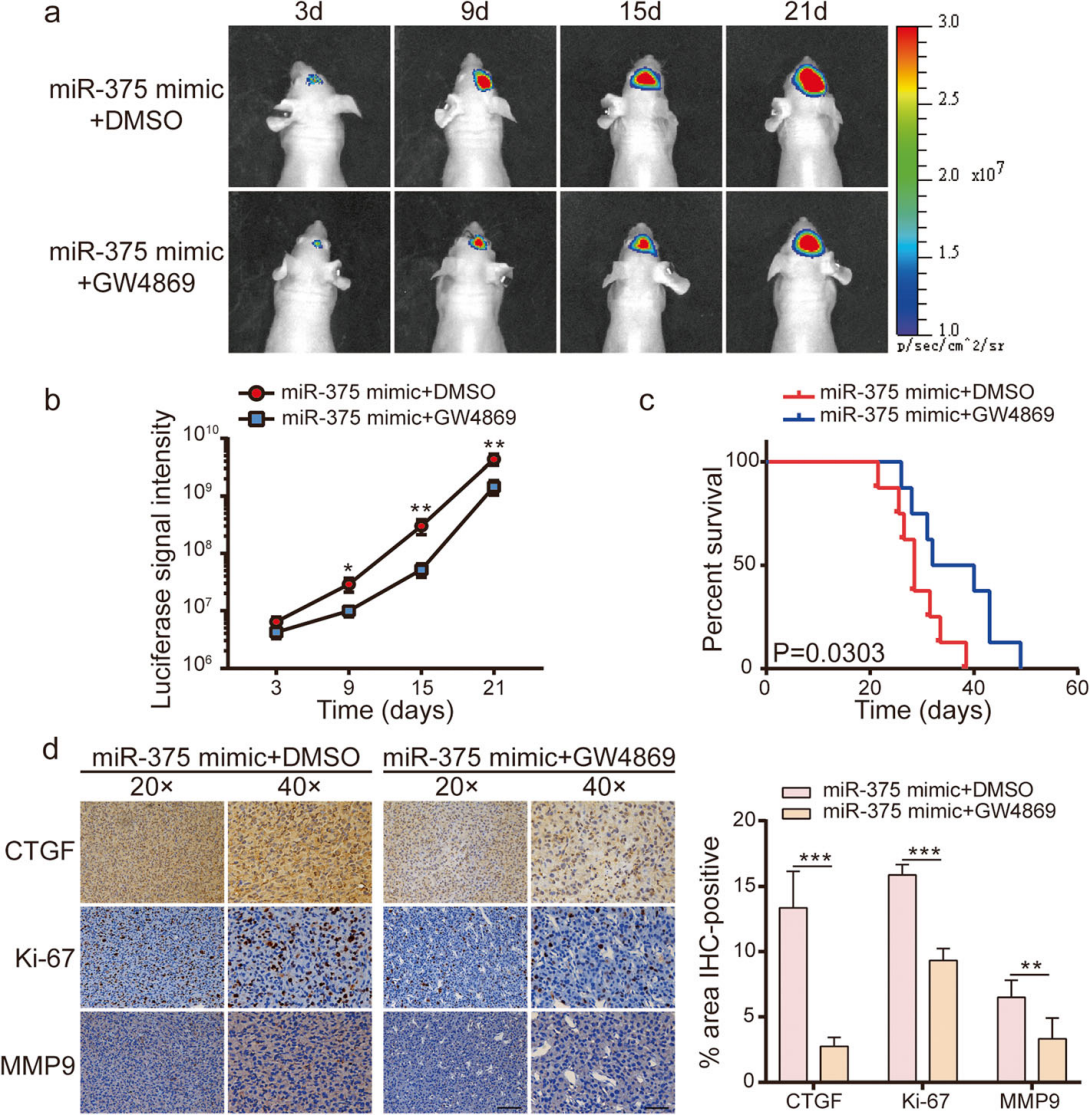
Inhibition of exosome secretion enhances miR-375 inhibition proliferation and invasion of glioma in vivo. **a.** Bioluminescence imaging indicating tumour size over time. **b.** Luminescent signal intensity of glioma-bearing mice in two groups. **c.** Evaluation of animal survival carried out according to Kaplan–Meier analysis. **d.** IHC staining of *CTGF*, Ki-67, and MMP9 in GW4869 group and DMSO group samples. Quantification of *CTGF*, Ki-67, and MMP9 intensity in IHC staining. Scale bar for 20X (left) = 100 μm, and 40X (right) =50 μm. Data are presented as mean ± standard deviation. *p < 0.05; **p < 0.01; ***p < 0.001. ns, not significant

To further investigate, in vivo, the pathophysiological significance of exosomal miR-375 release by glioma cells, a mouse model of orthotopic tumour transplantation was employed. First, U87-Luc-mimic cells were transplanted into the skull of nude mice to induce tumour formation. The mice were then intraperitoneally administered GW4869 at 1.25 μg/g body weight, three times per week starting on day 2 after surgical intervention until death. Control nude mice were injected with DMSO (i.e., the GW4869 solvent). We simultaneously repeated the animal experiment with G15-Luc-mimic cells. GW4869-treated mice displayed significantly reduced tumour size compared to control mice, as determined by bioluminescence imaging (Fig. 9a-b and Fig. S6a-b). Moreover, the GW4869 group exhibited a longer survival rate relative to controls (Fig. 9c). Lastly, *CTGF*, as well as the proliferation index marker, Ki-67, and the invasion marker, MMP9, were also decreased in GW4869-treated animals (Fig. 9d and Fig. S6c). These results demonstrated that suppression of exosome secretion exacerbated the inhibitory effects of miR-375 on proliferation and invasion of glioma in vivo.

## Discussion

**Fig. 10:**
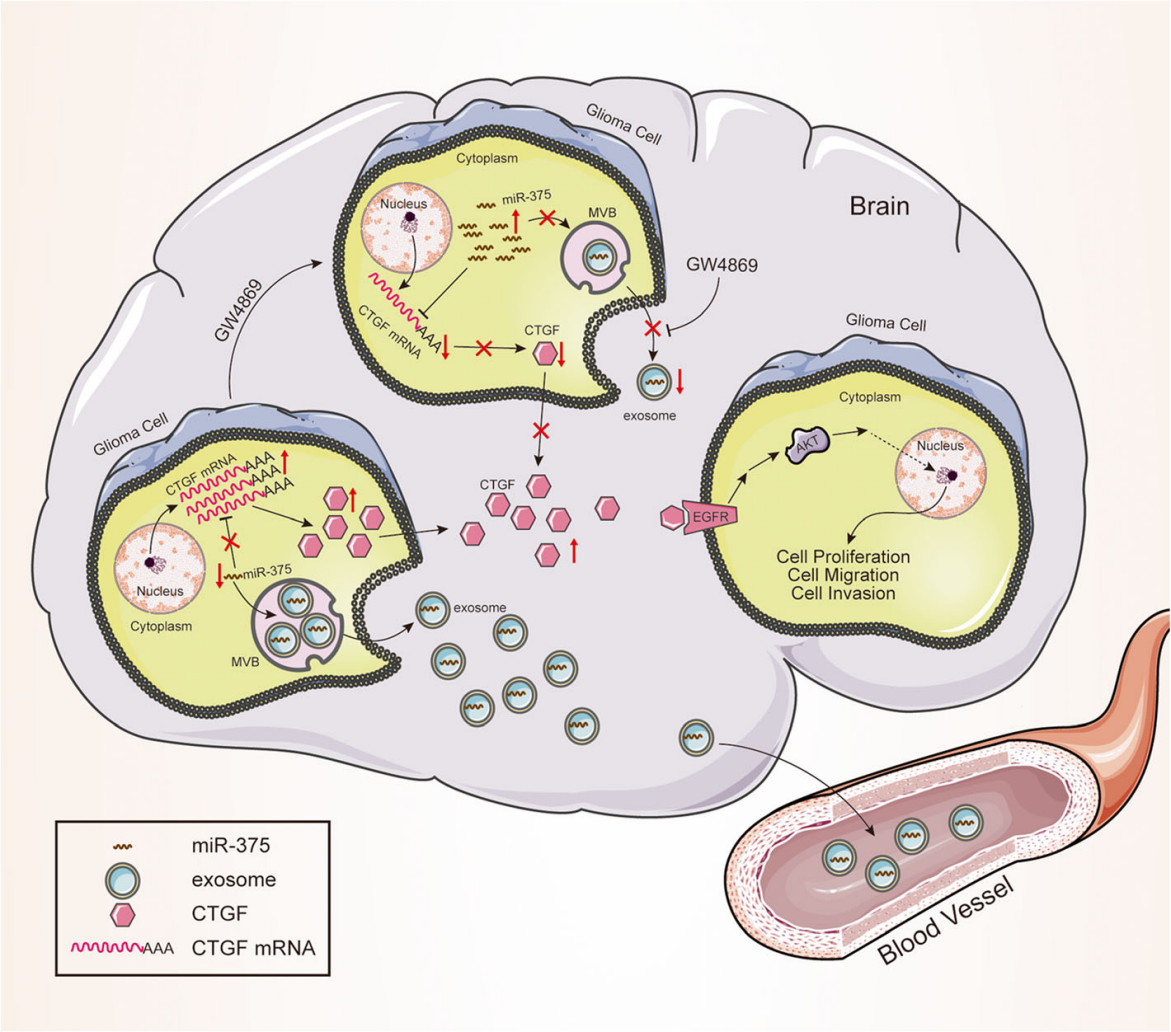
Schematic diagram depicting the molecular mechanism of exosome miR-375-mediated glioma cell proliferation, migration, and invasion. MiR-375 is excreted by glioma exosomes, which reduces the content of miR-375 in glioma cells. As a result, the inhibitory effect of miR-375 on *CTGF* is weakened. Therefore, glioma cells produce large amounts of *CTGF*. *CTGF* activates the *CTGF*-EGFR pathway of glioma cells through autocrine and paracrine methods. As a result, the proliferation, migration, and invasion of glioma cells are enhanced

This study reports three key findings. First, miR-375 is downregulated in glioma and has a role in the control of glioma cell proliferation and invasion. Second, the intracellular level and tumour-suppressive effects of miR-375 are reduced by exosomal secretion. Third, miR-375-containing exosomes released from glioma cells are not re-imported by glioma cells. Our results, therefore, identified a new mechanism by which glioma cells may eliminate tumour-suppressor miRNAs via exosome-mediated release (modelled in Fig. 10).

Here, using bioinformatics analysis, we found that miR-375 was poorly expressed in glioma tissues. This was confirmed by qRT-PCR, and was consistent with the results of previous studies [27, 28]. In particular, Chang et al. demonstrated that miR-375 expression is significantly lower in glioma tissues [27]. Similar results were recently reported by Gao and colleagues [28]. In addition, our results showed that miR-375 overexpression inhibited glioma *growth*, migration, and invasion, demonstrating that miR-375 exerted tumour-suppressive effects in glioma, confirming previous data obtained in other types of cancers, such as hepatocellular carcinoma, gastric cancer, oesophagus cancer, head and neck cancer [10], and colorectal cancer [29]. Specifically, Khondoker and co-workers found that miR-375 inhibits colorectal cancer cell proliferation and migration by targeting *CTGF* [29], demonstrating that the latter *factor* is a target of miR-375.

Therefore, we analysed the CGGA dataset and found that *CTGF* expression was upregulated in gliomas, and that its expression level was significantly inversely correlated with the prognosis of glioma patients, in line with previous studies on breast, prostate, glioma, pancreatic, and colon cancer, as well as thyroid carcinoma, chondrosarcoma, gallbladder carcinoma, melanoma, and leukaemia [37]. *CTGF* also has a role in the occurrence and development of various cancers by affecting extracellular matrix remodelling, angiogenesis, chemotaxis, cell adhesion and migration, as well as MMP expression [38]. Four public miRNA databases (TargetScan, Pictar, miRanda and StarBase) identified *CTGF* as a potential target of miR-375, while miR-375 was found to reduce *CTGF* expression in glioma cells. Notably, a previous study, using dual-luciferase assays, demonstrated that *CTGF* is a direct target of miR-375 [29]. Thus, we hypothesised that the same regulatory mechanism could also operate in gliomas.

EGFR, a membrane *receptor* with tyrosine kinase activity, is an important downstream effector of *CTGF* [39]. Upon *CTGF* binding, EGFR becomes phosphorylated and activates downstream signalling cascades [40]. EGFR is overexpressed in ~ 50–60% of gliomas [41], and its expression is positively correlated with malignancy grade. Furthermore, EGFR is required for the maintenance of glioma *growth* [42]; and the EGFR pathway participates in several cellular responses, such as proliferation, migration, cell differentiation, as well as in stability of the intracellular environment [43]. PI3K and Akt are downstream effectors of EGFR, while the EGFR-PI3K-Akt pathway is reportedly crucial for glioma progression [44]. In this study, we found that the *CTGF*-EGFR signalling pathway is affected by miR-375, *CTGF*, and EGF. Specifically, EGFR-AKT signalling was downregulated by miR-375 in U87, U251 and G15 cells, while the effect was clearly reversed by *CTGF* or EGF. These results identified miR-375 as a regulator of the *CTGF*-EGFR-AKT pathway and prompted us to study the function of the miR-375-*CTGF*-EGFR axis in glioma cells. We found that miR-375 regulated cell proliferation, migration, and invasion by controlling *CTGF*-EGFR signalling in glioma cells.

Evidence for the exosomal secretion of miR-375 by tumour cells, as well as for its potential applicability as a diagnostic marker for tumours, has been previously reported. Zhao and colleagues reported that exosomal miR-375 has an 85% accuracy in the detection of early-stage oestrogen *receptor*-positive breast cancer [12]. Moreover, Huang and co-workers showed that circulating exosomes containing miR-375 are promising prognostic biomarkers for castration-resistant prostate cancer patients [45]. In this study, qRT-PCR analysis demonstrated that miR-375 was secreted via exosomes by glioma cells. In addition, the level of miR-375 in purified exosomes was significantly higher compared to that of intracellular miR-375. This further demonstrates that miR-375 is actively secreted into exosomes. Notably, miR-375 was detected in circulating exosomes of glioma patients, with the level of exosomal miR-375 positively correlated with the glioma grade. These results indicate that circulating miR-375-containing exosomes can be used as a diagnostic marker for glioma. However, further experiments are required to verify this possibility.

The poor miR-375 expression in glioma cells relative to exosomes has been previously reported. Small miRNAs can be highly enriched in exosomes as a result of selective packaging [15, 21, 22]. Moreover, a crucial role of exosome release in the maintenance of cellular homeostasis has recently been reported. This task relies on the selective removal of harmful substances, such as DNA [46], proteins [47], and miRNAs from cells [24, 25]. Specifically, Akiko and colleagues reported that exosome secretion maintains cellular homeostasis by removing harmful cytoplasmic DNA from cells [46]. Meanwhile, Yun et al. found that the selective sorting of tumour-suppressor miR-193a into exosomes promotes colon cancer progression [24]. Moreover, the exosomal secretion of tumour-suppressor miR-23b was found to promote metastasis in bladder cancer [25]. However, prior to the present study, no evidence for the occurrence of this phenomenon in glioma had been presented.

The exosomal release of miRNAs relies on a ceramide-dependent secretion mechanism, and the biosynthesis of ceramide is regulated by nSMase2 [48]. Therefore, GW4869, a specific inhibitor of nSMase2, was used to suppress exosome secretion by glioma cells. Under these conditions, the intracellular level of miR-375 increased. In addition, the blockade of miR-375 exocytosis was associated with inhibition of glioma cell proliferation and invasion. These effects were associated with changes in the *CTGF*-EGFR signalling pathway.

Our study was the first to demonstrate that the selective sorting of tumour-suppressor miR-375 into exosomes promotes glioma progression. Therefore, in glioma, the inhibition of exosome secretion, combined with the use of traditional therapeutics, may result in increased antitumor efficacy. Notably, treatment of miR-375-overexpressing glioma cells with GW4869 enhanced the inhibitory effects of miR-375 on cell proliferation and invasion.

We also investigated whether glioma cells can re-import secreted miR-375-containing exosomes. Fluorescence microscopy and flow cytometry showed that miR-375-containing exosomes were poorly internalised by glioma cells, and, consistently, did not affect cell proliferation or invasion. These findings were confirmed by qRT-PCR. Hence, once released to the extracellular environment, miR-375-containing exosomes would no longer be imported by glioma cells and, therefore, would not affect their function. There are at least two possible explanations for this phenomenon. The first is that exosomes excreted as waste by glioma cells may express a ligand, e.g., CD47, that prevents their reuptake [49]. Alternatively, these exosomes may lack a specific ligand required for the recognition and internalisation by glioma cells. This issue may be addressed by mass spectrometry analysis of the proteins present on the exosomal surface. Notably, in glioma, the therapeutic effect of exosomes serving as drug carriers was found to be enhanced by the modification of specific ligands located on the exosomal surface.

Our work suggests that exosomal release is a way by which glioma cells may eliminate miRNAs. We found that the exosomal secretion of the tumour-suppressor miR-375 by glioma cells leads to activation of the *CTGF*-EGFR oncogenic pathway, thus promoting glioma proliferation and invasion (Fig. 10). Further studies are needed to characterise the machinery responsible for the selective exosomal packaging of miRNAs.

## Conclusions

The removal of tumour-suppressor miR-375 from glioma cells via exosome secretion ensures the sustained activation of the *CTGF*-EGFR carcinogenic pathway, which promotes the proliferation, migration, and invasion of glioma. This study, therefore, serves to advance the current understanding of exosome biology and provide new directions for the treatment of gliomas, suggesting that exosomal miR-375 may be a potential clinical biomarker for glioma.

## Supplementary Information

**Additional file 1: Figure S1** MiR-375 inhibits glioma progression in vivo. **a.** MiR-375 overexpression in U87-Luc cells validated by qRT-PCR. **b.** Luminescent imaging of representative nude mice from U87-Luc cells transfected with miR-375 mimic (*n* = 8) or miR-control (*n =* 8) lentiviruses at day 3, 9, and 15. **c.** Luminescent signal intensity of the glioma-bearing mice in two groups. **d.** IHC staining of Ki-67 in MiR-375 overexpression or control tumour samples. Scale bar for 20X (upper panel): 100 μm and 40X (lower panel) = 50 μm. **e.** H&E staining images showing the junctions between glioma xenografts and surrounding brain tissues. Scale bar = 100 μm. Data are presented as mean ± standard deviation. **p < 0.01; ***p < 0.001. ns, not significant.

**Additional file 2: Figure S2** miR-375 regulates the proliferation and invasion of the primary glioma cell line, G15, through the *CTGF*-EGFR signalling pathway. **a.** qRT-PCR assay demonstrating expression of miR-375 in G15 cells compared to HEB cells. **b.** MiR-375 overexpression in G15 cells validated by qRT-PCR. **c.** Expression of candidate target gene *CTGF* in G15 cells assessed by qRT-PCR following overexpression of miR-375. **d.** Western blot analysis of *CTGF*, p-EGFR (Tyr1068), EGFR, p-AKT (Ser-473), AKT, and MMP9 in G15 cells. **e.** CCK-8 analysis detected effects of *CTGF* or EGF addition on the *growth* inhibitory effect of miR-375. **f.** Transwell analysis demonstrating the effect of *CTGF* or EGF addition on the inhibitory effect of miR-375 on G15 cell migration (upper panel) and invasion (lower panel). Scale bar = 100 μm. All experiments were repeated independently three times. Data are presented as mean ± standard deviation. **p < 0.01; ***p < 0.001. ns, not significant.

**Additional file 3: Figure S3** G15 cell-derived exosomes carry a high expression of miR-375. **a.** Immunoblotting for exosomal markers, ALIX, TSG101 and CD9, as well as negative control, calnexin. **b.** Uranyl acetate negative stained TEM images, of exosomes isolated from G15 cells. Scale bar, 200 nm. **c.** Size distribution of the isolated exosomes analysed by Zetasizer Nano-Zs. **d.** Detection of miR-375 relative expression levels in G15 cells and their derived exosomes by qRT-PCR. All experiments were repeated independently three times. Data are presented as mean ± standard deviation. ***p < 0.001.

**Additional file 4: Figure S4** Regulates the secretion of exosomes. **a.** Total protein content in exosomes after treatment with 10 μM GW4869. **b.** Western blot analysis of ALIX and TSG101 in exosomes secreted by U87, U251, and G15 cells treated with 10 μM GW4869. **c.** Total protein content in exosomes after treatment with 1 μM monensin. **d.** Western blot analysis of ALIX and TSG101 in exosomes secreted by U87 and U251 cells treated with 1 μM monensin. All experiments were repeated independently three times. Data are presented as mean ± standard deviation. **p < 0.01; ***p < 0.001. ns, not significant.

**Additional file 5: Figure S5** Exosomes regulate the proliferation and invasion of G15 cells through the miR-375-*CTGF*-EGFR signalling pathway. **a.** Changes in miR-375 levels in miR-375 overexpressing G15 cells treated with or without GW4869 examined by qRT-PCR. **b.** Effect of GW4869 on the inhibitory effect of miR-375 against G15 cells proliferation, as determined using CCK-8 analysis. **c.** Transwell analysis demonstrating the effect of GW4869 on the inhibitory effect of miR-375 against glioma cell migration (upper panel) and invasion (lower panel). Scale bar = 100 μm. **d.** Western blot analysis of *CTGF*, p-EGFR (Tyr1068), EGFR, p-AKT (Ser-473), AKT, and MMP9 in G15 cells. All experiments were repeated independently three times. Data are presented as mean ± standard deviation. *p < 0.05; **p < 0.01; ***p < 0.001. ns, not significant.

**Additional file 6: Figure S6** Inhibition of exosome secretion enhances miR-375 inhibition proliferation and invasion of gliomas formed by G15 in vivo. **a.** Bioluminescence imaging depicting tumour size over time. **b.** The luminescent signal intensity of the glioma-bearing mice in two groups. **c.** IHC staining of *CTGF*, Ki-67, and MMP9 in the GW4869 and DMSO group samples. Quantification of *CTGF*, Ki-67, and MMP9 intensity via IHC staining. Scale bar for 20X (left) =100 μm and 40X (right) = 50 μm. Data are presented as mean ± standard deviation. *p < 0.05; **p < 0.01; ***p < 0.001. ns, not significant.

**Additional file 7: Table S1.** List of primary antibodies.

## Data Availability

The datasets during and/or analyzed during the current study are available from the corresponding author on reasonable request.

## Abbreviations

WHO: World Health Organization
GBM: Glioblastoma multiforme
miRNAs: microRNAs
3′-UTR: 3′-untranslated region
miR-375: microRNA-375
*CTGF*: *Connective* *tissue* *growth* *factor*
EGFR: *Epidermal* *growth* *factor* *receptor*
dbDEMC: Database of Differentially Expressed MiRNAs in human Cancers
ATCC: American Type Culture Collection
DMEM: Dulbeccos modified Eagles medium
FBS: Fetal bovine serum
qRT-PCR: Quantitative real-time polymerase chain reaction
EdU: 5-ethynyl-2′-deoxyuridine
DAPI: 4′,6-diami-dino-2-phenylindole
ECL: Enhanced chemiluminescence
ELISA: Enzyme linked immunosorbent assay
TEM: Transmission electron microscopy
BCA: Bicinchoninic acid
H&E: Hematoxylin and eosin
IHC: Immunohistochemistry
DAB: 3,3′-diaminobenzidine
nSMase2: Neutral sphingomyelinase 2
ADE: Astrocyte-derived exosome
BNCA: Brain cancer
GLAST: Glutamine aspartate transporter
hMSC: Human marrow stromal cells
