# Supplementary figures and images for: Selective exosome exclusion of miR-375 by glioma cells promotes glioma progression by activating the CTGF-EGFR pathway
Source: J Exp Clin Cancer Res. 2021 Jan 6;40:16. doi: 10.1186/s13046-020-01810-9 (PMC7789663; doi:10.1186/s13046-020-01810-9)

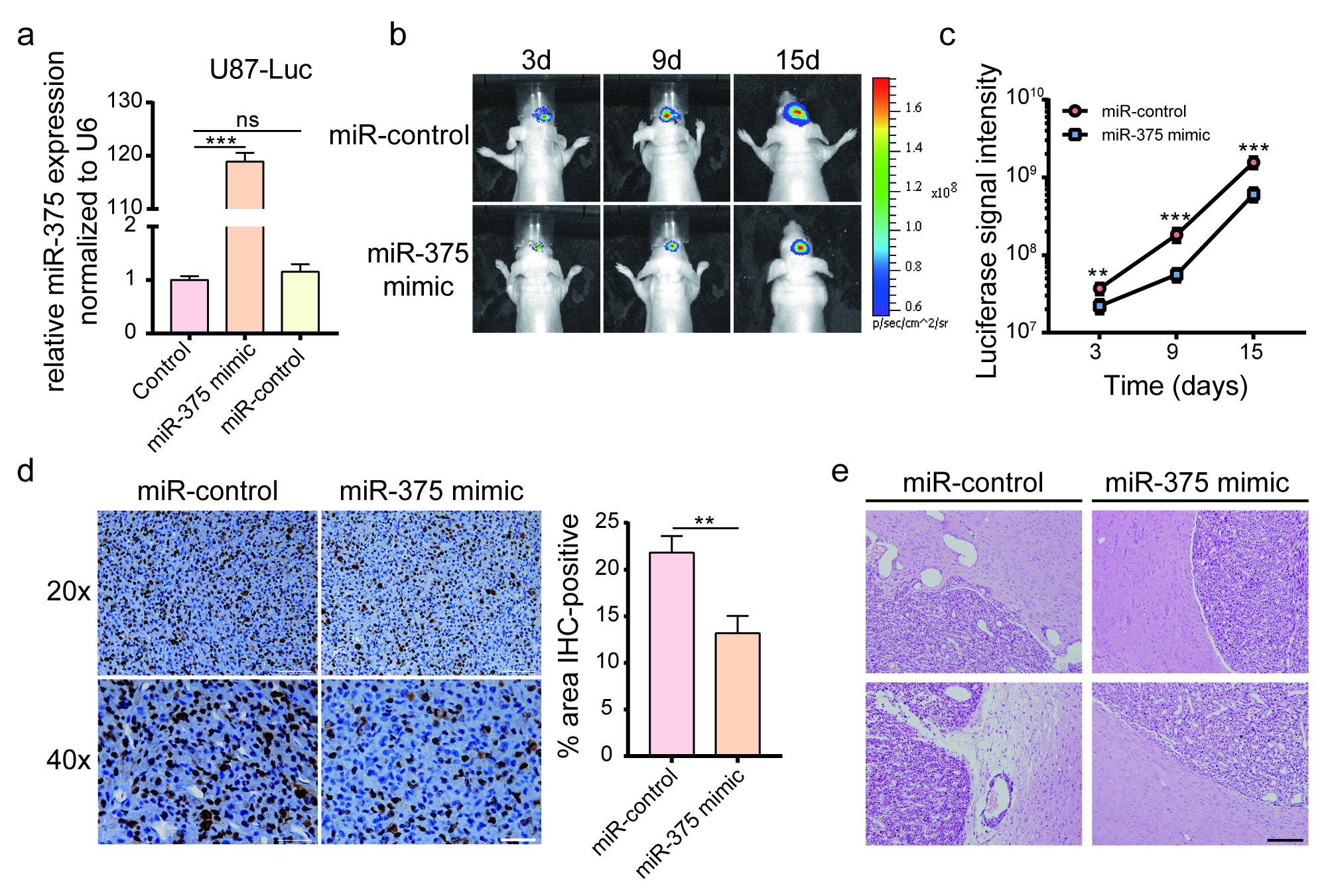

Supplement: Supplementary file 1 — Additional file 1: Figure S1 MiR-375 inhibits glioma progression in vivo. a. MiR-375 overexpression in U87-Luc cells validated by qRT-PCR. b. Luminescent imaging of representative nude mice from U87-Luc cells transfected with miR-375 mimic (n = 8) or miR-control (n = 8) lentiviruses at day 3, 9, and 15. c. Luminescent signal intensity of the glioma-bearing mice in two groups. d. IHC staining of Ki-67 in MiR-375 overexpression or control tumour samples. Scale bar for 20X (upper panel): 100 μm and 40X (lower panel) = 50 μm. e. H&E staining images showing the junctions between glioma xenografts and surrounding brain tissues. Scale bar = 100 μm. Data are presented as mean ± standard deviation. **p < 0.01; ***p < 0.001. ns, not significant. [file 13046_2020_1810_MOESM1_ESM.tif]

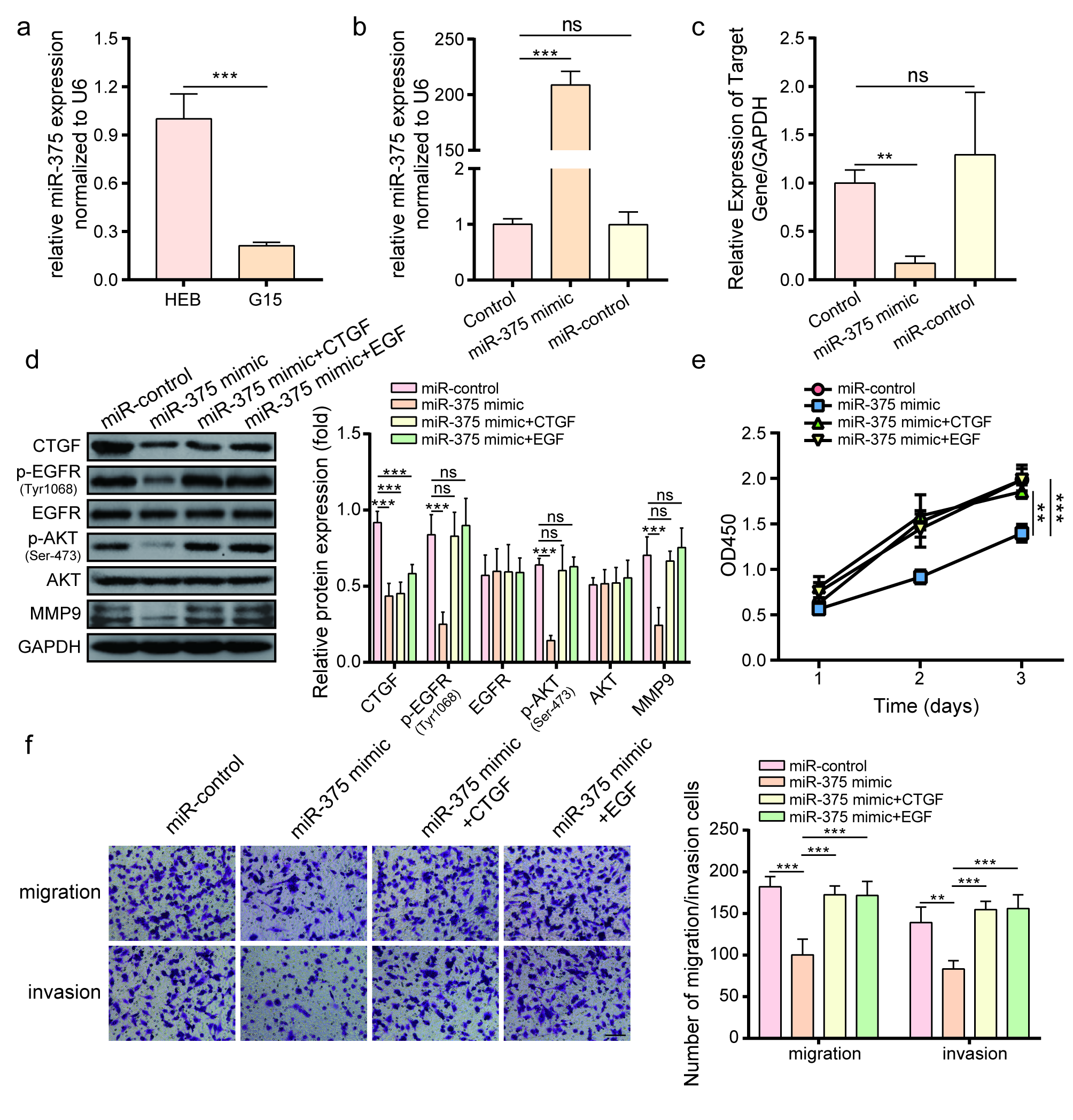

Supplement: Supplementary file 2 — Additional file 2: Figure S2 miR-375 regulates the proliferation and invasion of the primary glioma cell line, G15, through the CTGF-EGFR signalling pathway. a. qRT-PCR assay demonstrating expression of miR-375 in G15 cells compared to HEB cells. b. MiR-375 overexpression in G15 cells validated by qRT-PCR. c. Expression of candidate target gene CTGF in G15 cells assessed by qRT-PCR following overexpression of miR-375. d. Western blot analysis of CTGF, p-EGFR (Tyr1068), EGFR, p-AKT (Ser-473), AKT, and MMP9 in G15 cells. e. CCK-8 analysis detected effects of CTGF or EGF addition on the growth inhibitory effect of miR-375. f. Transwell analysis demonstrating the effect of CTGF or EGF addition on the inhibitory effect of miR-375 on G15 cell migration (upper panel) and invasion (lower panel). Scale bar = 100 μm. All experiments were repeated independently three times. Data are presented as mean ± standard deviation. **p < 0.01; ***p < 0.001. ns, not significant. [file 13046_2020_1810_MOESM2_ESM.tif]

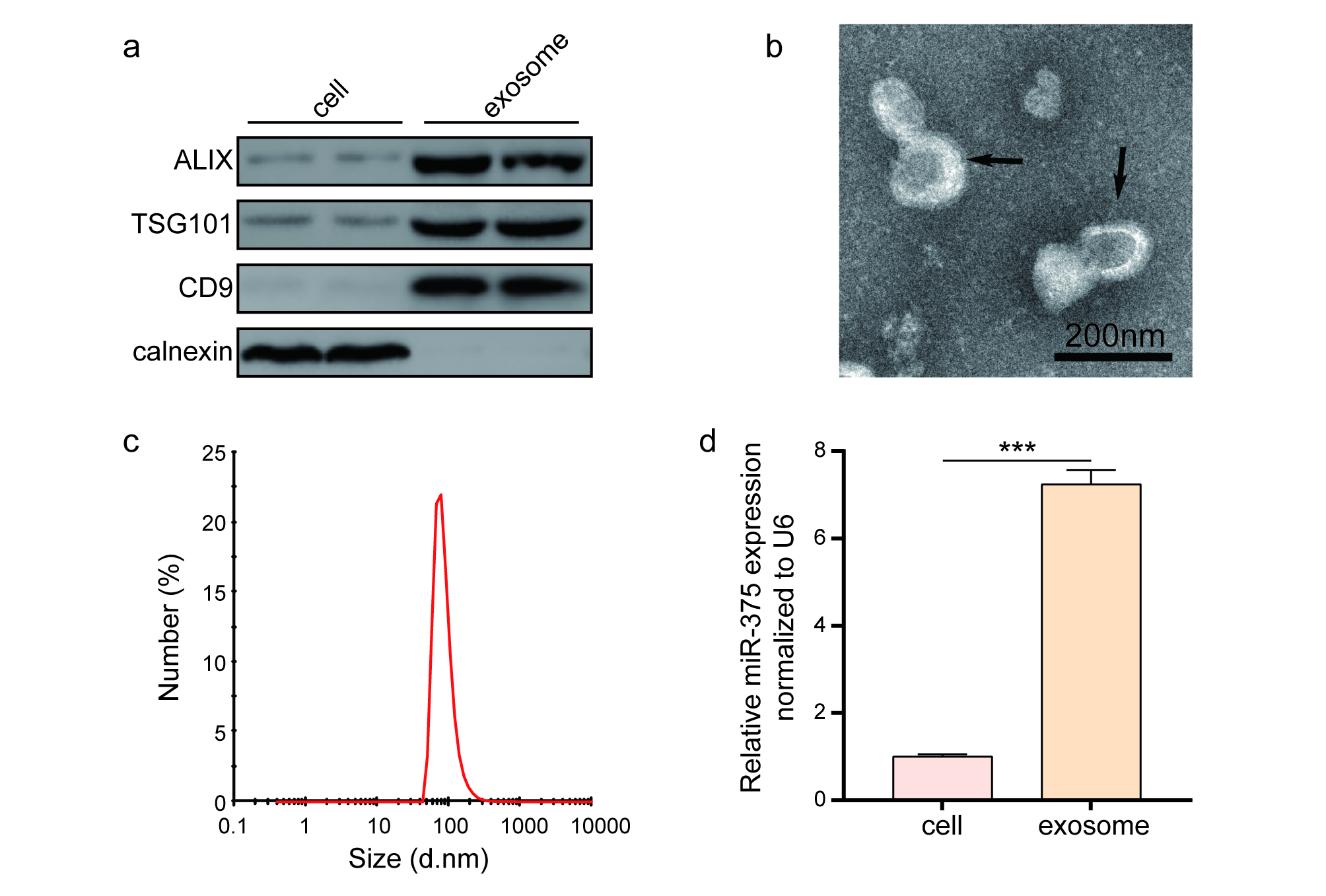

Supplement: Supplementary file 3 — Additional file 3: Figure S3 G15 cell-derived exosomes carry a high expression of miR-375. a. Immunoblotting for exosomal markers, ALIX, TSG101 and CD9, as well as negative control, calnexin. b. Uranyl acetate negative stained TEM images, of exosomes isolated from G15 cells. Scale bar, 200 nm. c. Size distribution of the isolated exosomes analysed by Zetasizer Nano-Zs. d. Detection of miR-375 relative expression levels in G15 cells and their derived exosomes by qRT-PCR. All experiments were repeated independently three times. Data are presented as mean ± standard deviation. ***p < 0.001. [file 13046_2020_1810_MOESM3_ESM.tif]

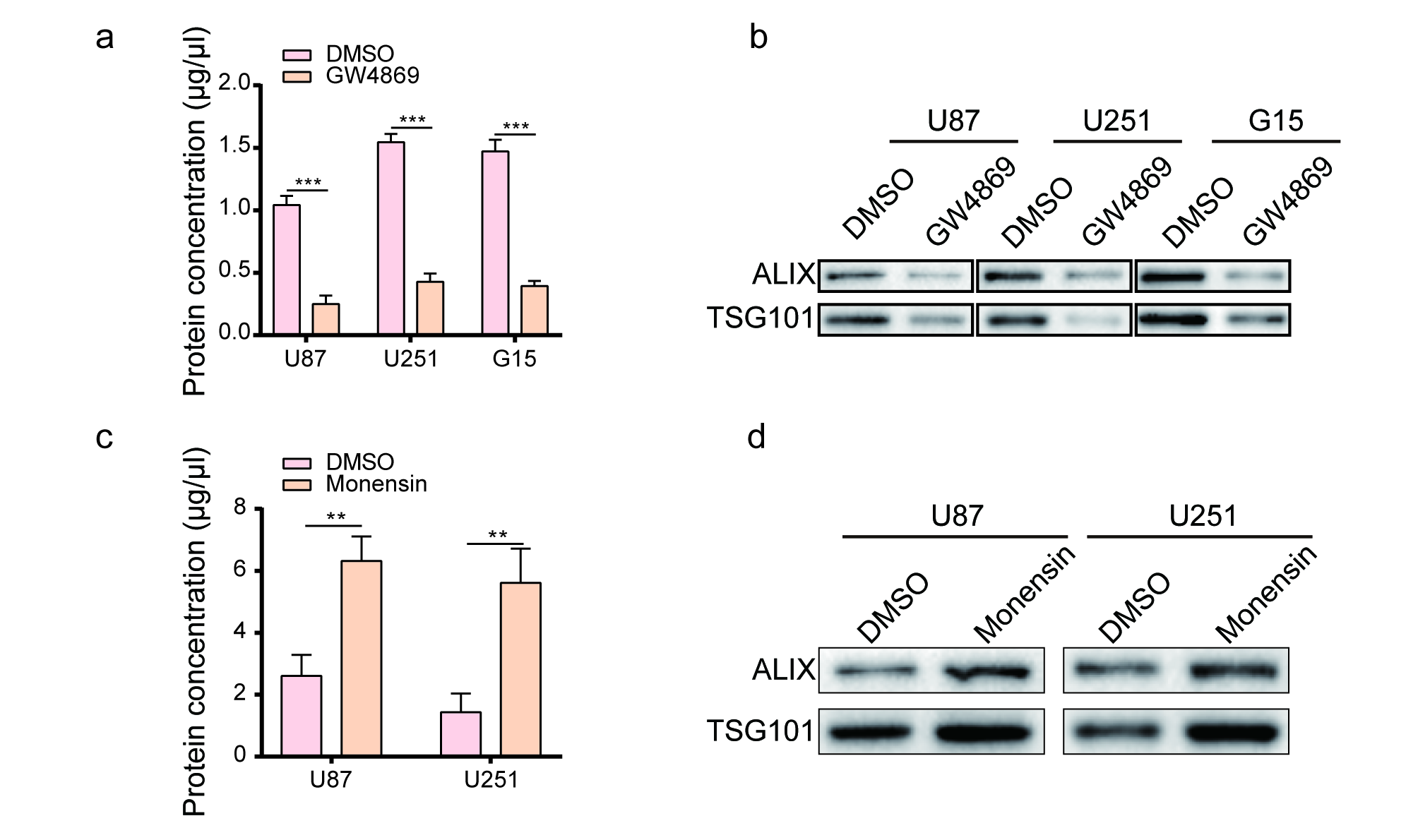

Supplement: Supplementary file 4 — Additional file 4: Figure S4 Regulates the secretion of exosomes. a. Total protein content in exosomes after treatment with 10 μM GW4869. b. Western blot analysis of ALIX and TSG101 in exosomes secreted by U87, U251, and G15 cells treated with 10 μM GW4869. c. Total protein content in exosomes after treatment with 1 μM monensin. d. Western blot analysis of ALIX and TSG101 in exosomes secreted by U87 and U251 cells treated with 1 μM monensin. All experiments were repeated independently three times. Data are presented as mean ± standard deviation. **p < 0.01; ***p < 0.001. ns, not significant. [file 13046_2020_1810_MOESM4_ESM.tif]

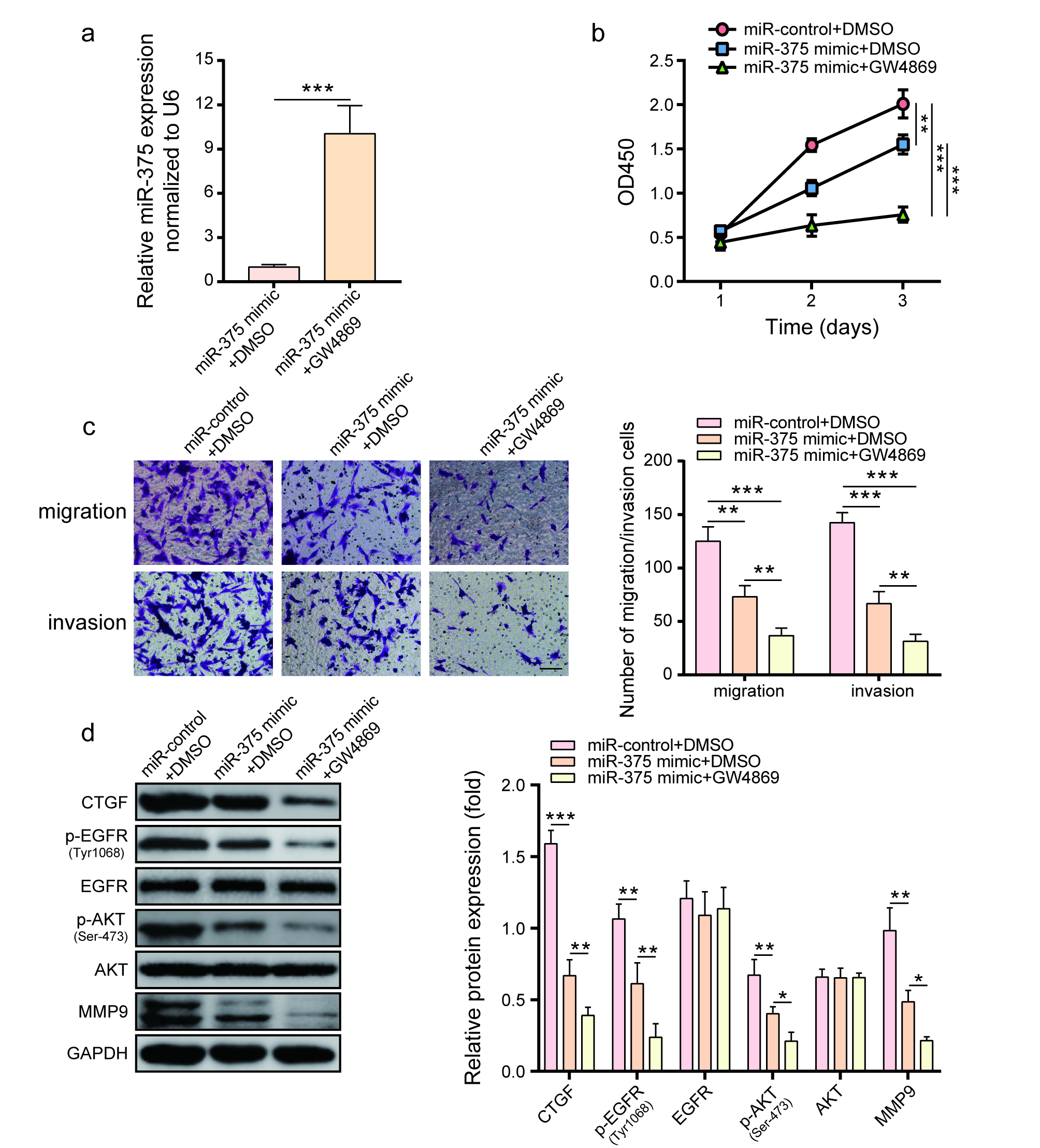

Supplement: Supplementary file 5 — Additional file 5: Figure S5 Exosomes regulate the proliferation and invasion of G15 cells through the miR-375-CTGF-EGFR signalling pathway. a. Changes in miR-375 levels in miR-375 overexpressing G15 cells treated with or without GW4869 examined by qRT-PCR. b. Effect of GW4869 on the inhibitory effect of miR-375 against G15 cells proliferation, as determined using CCK-8 analysis. c. Transwell analysis demonstrating the effect of GW4869 on the inhibitory effect of miR-375 against glioma cell migration (upper panel) and invasion (lower panel). Scale bar = 100 μm. d. Western blot analysis of CTGF, p-EGFR (Tyr1068), EGFR, p-AKT (Ser-473), AKT, and MMP9 in G15 cells. All experiments were repeated independently three times. Data are presented as mean ± standard deviation. *p < 0.05; **p < 0.01; ***p < 0.001. ns, not significant. [file 13046_2020_1810_MOESM5_ESM.tif]

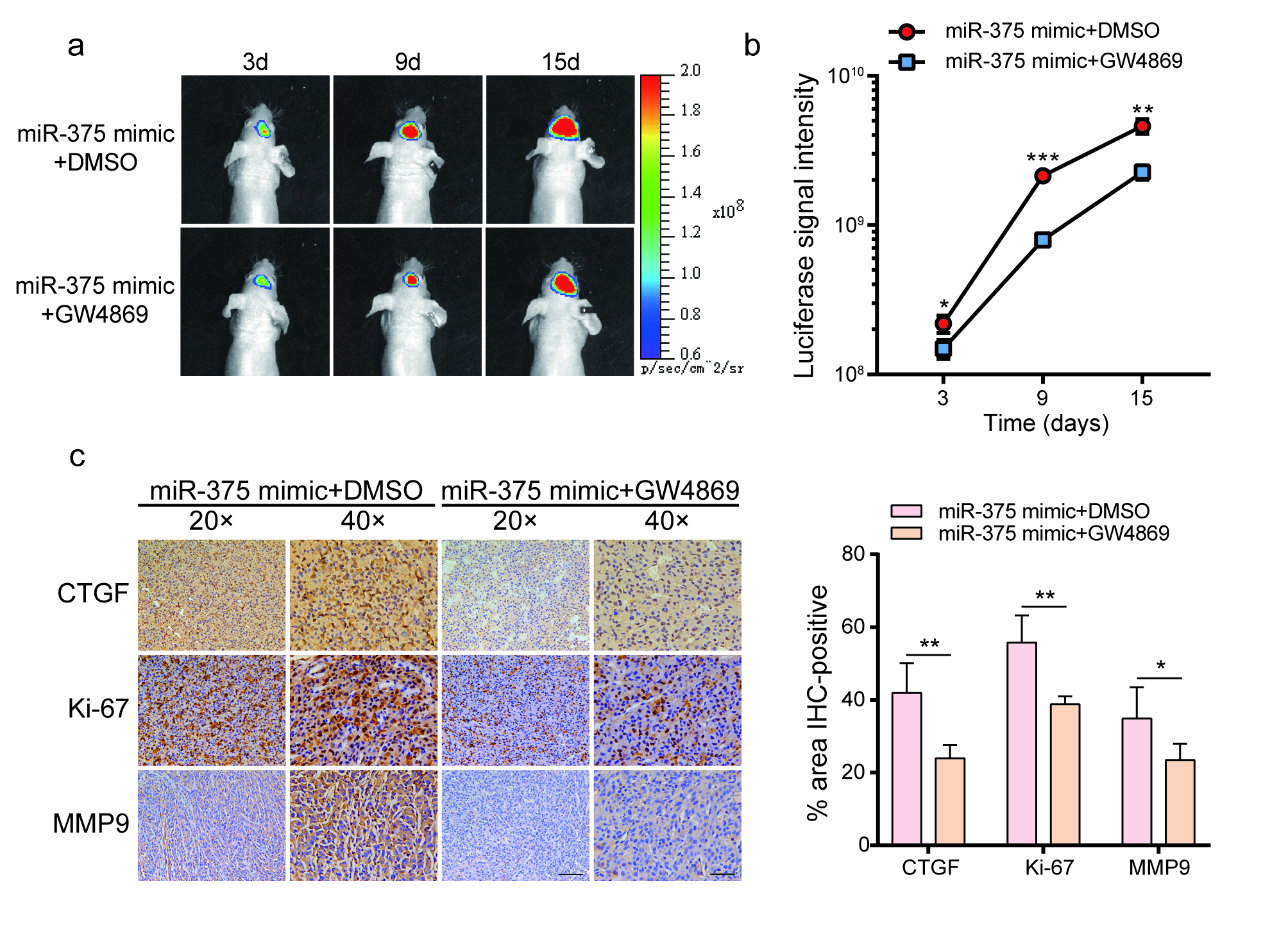

Supplement: Supplementary file 6 — Additional file 6: Figure S6 Inhibition of exosome secretion enhances miR-375 inhibition proliferation and invasion of gliomas formed by G15 in vivo. a. Bioluminescence imaging depicting tumour size over time. b. The luminescent signal intensity of the glioma-bearing mice in two groups. c. IHC staining of CTGF, Ki-67, and MMP9 in the GW4869 and DMSO group samples. Quantification of CTGF, Ki-67, and MMP9 intensity via IHC staining. Scale bar for 20X (left) =100 μm and 40X (right) = 50 μm. Data are presented as mean ± standard deviation. *p < 0.05; **p < 0.01; ***p < 0.001. ns, not significant. [file 13046_2020_1810_MOESM6_ESM.tif]
